# Attenuation of cGAS-STING signaling-mediated lung inflammation during infection through autophagy induction by bioactive nanodevices

**DOI:** 10.7150/thno.127113

**Published:** 2026-03-04

**Authors:** Mimi Pang, Xiang Wang, Zichen Song, Rujing Lin, Sixia Liu, Man Xing, Wenfei Xu, Jiameng Gong, Ying Qi, Mei Du, Yonghao Yu, Bing Chen, Shan-Yu Fung, Dongming Zhou, Hong Yang

**Affiliations:** 1 Department of Pharmacology and Tianjin Key Laboratory of Inflammation Biology, The Province and Ministry Co-Sponsored Collaborative Innovation Center for Medical Epigenetics, School of Basic Medical Sciences, Intensive Care Unit of the Second Hospital, Tianjin Medical University, Tianjin 300070, China.; 2 Shanghai Public Health Clinical Center, Fudan University, Shanghai 201508, China.; 3 Department of Anesthesia, Tianjin Institute of Anesthesiology, Tianjin Medical University General Hospital, Tianjin 300052, China.; 4 Department of Pathogen Biology, School of Basic Medical Sciences, Tianjin Medical University, Tianjin 300070, China.; 5 Institute of Infectious Diseases, Intensive Care Unit of the Second Hospital, Tianjin Medical University, Tianjin 300211, China.; 6 State Key Laboratory of Experimental Hematology, Department of Immunology and Key Laboratory of Immune Microenvironment and Disease (Ministry of Education), School of Basic Medical Sciences, Tianjin Medical University, Tianjin 300070, China.; 7 International Joint Laboratory of Ocular Diseases (Ministry of Education), Tianjin Key Laboratory of Ocular Trauma, Tianjin Medical University, Tianjin 300052, China.

**Keywords:** bioactive nanodevice, cGAS-STING, viral pneumonia, macrophage, autophagy

## Abstract

**Background:**

Modulating the cGAS-STING pathway by bioactive nanodevices is a promising strategy for combating infection-associated inflammatory disorders. However, the development of pharmacological inhibitors for cGAS-STING signaling is currently hindered by lacking cell-specific targeting capability. This study aimed to develop a potent, drug-free nanodevice that specifically targets pulmonary macrophages to modulate the cGAS-STING pathway for ameliorating infection-associated detrimental lung inflammation.

**Methods:**

Cigarette smoke extract-modified peptide gold nanoparticle hybrids (CSE-P12) were synthesized. Transcriptomic analysis, western blotting, autophagy reporter assays, and confocal microscopy were employed to assess the effects of CSE-P12 on gene expression, STING degradation, autophagic flux, and inflammation. TEM imaging and LC-MS/MS were utilized to elucidate the molecular mechanisms underlying CSE-P12-induced autophagy in macrophages. Finally, the HAdV4-induced pneumonia and CLP-induced sepsis models on wild-type and STING^-/-^ mice were used to evaluate the therapeutic efficacy of CSE-P12 and validate its inhibitory mechanisms on the cGAS-STING pathway.

**Results:**

CSE-P12 nanodevices are extensively internalized by macrophages via energy-dependent cellular uptake. This large internalization triggers autophagic degradation of STING, thereby effectively inhibiting the cGAS-STING-mediated interferon responses and inflammation. In the HAdV4-induced viral pneumonia mouse model, intratracheally instilled CSE-P12 effectively targets pulmonary macrophages, suppresses STING activation, and significantly alleviates lung inflammation and injury. The depletion of the pulmonary macrophages abolishes these protective effects. The therapeutic potential of CSE-P12 is further validated in a CLP-induced polymicrobial sepsis mouse model, where it significantly prolongs mouse survival and decreases lung inflammation.

**Conclusions:**

CSE-P12 effectively targets pulmonary macrophages and exhibits potent anti-inflammatory activities in viral pneumonia and sepsis-induced acute lung injury by inducing autophagic flux to facilitate STING degradation. This work provides a new paradigm for designing targeted nanotherapeutics to modulate STING activation in inflammatory diseases.

## Introduction

As lungs possess the largest interface vulnerable to foreign pathogens, they rely on a delicate immune surveillance system for the rapid clearance of invading microbes 1. The pulmonary immune system utilizes a variety of robust and sensitive recognition mechanisms to identify distinct extracellular and intracellular molecular patterns. The membrane-bound pattern recognition receptors (PRRs), such as Toll-like receptors (TLRs), provide first-line sensing of extracellular pathogens, while cytosolic sensors, like the cyclic GMP-AMP synthase (cGAS), recognize the intracellular threats 2. Particularly, the evolutionarily conserved cGAS- stimulator of interferon genes (STING) sensing mechanism has garnered significant attention for its critical role in detecting aberrant cytosolic DNA from invading pathogens or diseased host cells 3. Upon DNA recognition, cGAS catalyzes the formation of 2′3′-cyclic-GMP-AMP (cGAMP), which activates STING on the endoplasmic reticulum (ER). This subsequently triggers TANK-binding kinase 1 (TBK1)-mediated signaling cascades to drive interferon (IFN) regulatory factor 3 (IRF3) and nuclear factor-κB (NF-κB) activation for the production of type I IFNs and proinflammatory cytokines 4. Although this signaling pathway is indispensable for antiviral immunity 5, emerging clinical evidence reveals that sustained STING hyperactivation has become a pathological hallmark in over 50% of severe COVID-19 pneumonia patients and 45% of sepsis-induced acute lung injury (ALI) cases, correlating with elevated IFN-β levels and poor patient outcomes 6-8. These facts highlight the urgent yet challenging need to selectively suppress the pathological STING hyperactivity in the lung without compromising the surveillance function of the cGAS-STING pathway in pathogen clearance.

Pulmonary macrophages are the predominant sentinel immune cells in the lung, possessing a heightened capability to sense and respond to foreign cytosolic DNA via cGAS-STING activation to mount pulmonary inflammation during infection 9, 10. However, studies indicate that macrophage-specific STING activation often leads to overwhelming inflammatory responses that exacerbate lung injuries 11. For example, infection with carbapenem-resistant *Acinetobacter baumannii* (CRAB) triggers the release of mitochondrial DNA to activate cGAS-STING pathway in macrophages and aggravate lung inflammation 12. Conversely, the STING-specific inhibitor gelsevirine attenuates sepsis-induced lung injury in mice by locking STING in an inactive conformation and promoting its proteasomal degradation 13. Thus, the targeted inhibition of pathogenic cGAS-STING activation in macrophages may represent a promising therapeutic strategy to control excessive, infection-mediated pulmonary inflammation.

Numerous small molecule inhibitors (SMIs) have been developed to modulate cGAS-STING signaling 14. These SMIs are generally classified into two categories based on their distinct mechanisms of action. One class consists of covalent inhibitors (e.g., C-170, C-171, C-176, C-178, and H-151), which covalently bind to the Cys88/91 residues of STING. This engagement blocks STING palmitoylation, a step essential for STING trafficking from the ER to the Golgi 15. The other includes the competitive C-terminal domain antagonists (e.g., Astin C and compound 18), which compete with cGAMP for the binding pocket at the C-terminal domain of STING, thereby preventing its conformational change required for TBK1 recruitment 16. Although these SMIs have demonstrated efficacy in murine models of autoimmune disease, their clinical translation is impeded by several pharmacological barriers, including low potency, off-target effects, poor cell selectivity, and limited bioavailability in target tissues. To address these challenges, we aimed to develop a potent drug-free nanodevice that specifically targets pulmonary macrophages to modulate the cGAS-STING pathway and manage infection-associated detrimental lung inflammation.

Recent studies have revealed that the STING level can be regulated through autophagy-mediated degradation 17. Upon activation, STING interacts with the microtubule-associated protein 1 light chain 3 (LC3) to trigger autophagy independently of TBK1 activation 17; in contrast, TBK1-phosphorylated Sequestosome 1 (SQSTM1 or p62), the selective autophagy receptor, can facilitate the autophagosome degradation of STING 18. These endogenous regulatory mechanisms inspired a paradigm-shifting strategy: utilizing engineered nanodevices to induce autophagy-lysosomal degradation of STING in macrophages to down-regulate pathogenic cGAS-STING signaling. Our previous work demonstrated that cigarette smoke extract (CSE)-modified peptide gold nanoparticle hybrids (CSE-P12) effectively induce protective autophagy, mitigating inflammatory responses in macrophages 19. However, it remains unknown whether CSE-P12 could specifically attenuate the cGAS-STING-mediated inflammatory signaling by inducing the autophagic degradation of STING for controlling infection-associated lung inflammation and injuries. Furthermore, the precise molecular mechanism by which CSE-P12 induces autophagy in macrophages warrants further investigation.

**Scheme 1:**
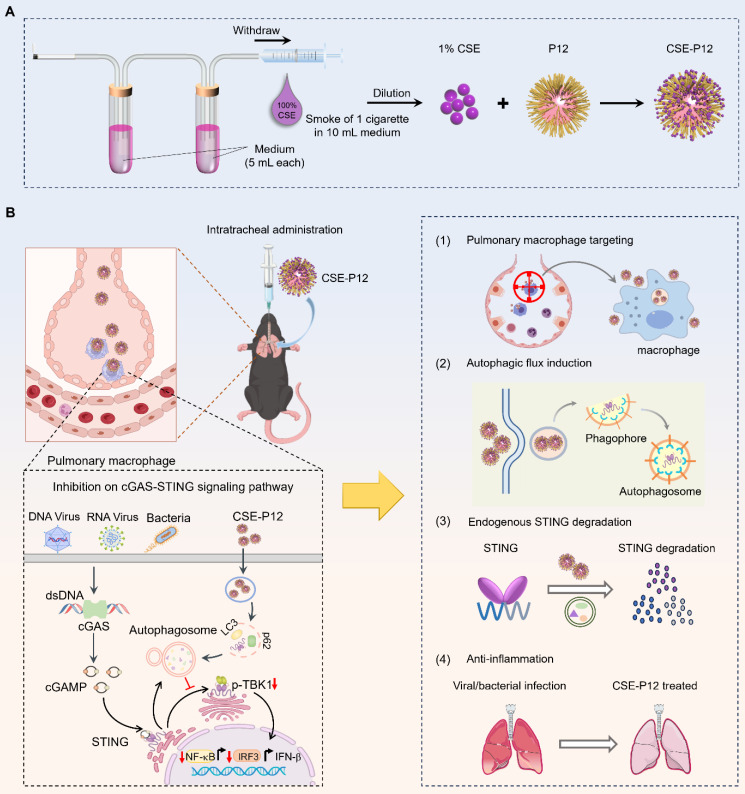
** Precision nanotherapeutic strategy for pneumonia intervention by targeted inhibition of cGAS-STING signaling pathway in pulmonary macrophages.** (A) The fabrication of the cigarette smoke extract (CSE)-modified peptide-gold nanoparticles CSE-P12. (B) The proposed mechanism of the therapeutic effects of CSE-P12 on pneumonia under viral/bacterial infection with four highlighted features: pulmonary macrophage targeting, autophagic influx induction, effective STING degradation, and potent anti-inflammatory activities.

Herein, we aim to investigate the novel activities of CSE-P12 in modulating the cGAS-STING signaling pathway and its therapeutic efficacy in viral pneumonia and sepsis-induced ALI (**Scheme 1**). First, the unbiased global transcriptome analysis was conducted to define the regulatory spectrum of CSE-P12 on STING activation in macrophages. Next, the CSE-P12-induced autophagy and the subsequent autophagy-lysosomal degradation of STING were examined in macrophages. To evaluate the therapeutic efficacy of CSE-P12 and clarify its acting mechanism via inhibiting the cGAS-STING pathway, the adenovirus-induced pneumonia and cecal ligation and puncture (CLP)-induced sepsis models were employed in both wild-type and STING^-/-^ mice. The macrophage-dependent efficacy was further validated through macrophage depletion by clodronate liposomes in the viral pneumonia mouse model. Mechanistically, we discovered that the enormous uptake of CSE-P12 in macrophages triggered ADP/ATP-dependent activation of AMP-activated protein kinase (AMPK) to induce autophagic degradation of STING. Our results demonstrate that CSE-P12 possesses four unique therapeutic features: (i) specific targeting of pulmonary macrophages, ii) potent induction of autophagic flux, (iii) facilitation of STING degradation, and (iv) significant anti-inflammatory activity (**Scheme 1B**). This study establishes a novel strategy for designing autophagy-inducing nanodevices to control lung inflammation driven by excessive cGAS-STING activation.

## Materials and Methods

### Materials

Wild-type human adenovirus 4 (HAdV4) and A/Puerto Rico/8/34 (PR8) virus were preserved in Dongming Zhou's laboratory. Hexapeptides were synthesized from Nanjing Jietai Biological Company (Nanjing, China). 2'3'-cGAMP (tlrl-nacga23-2) was obtained from InvivoGen (San Diego, CA, USA). 3-methyladenine (3-MA) (HY-19312), compound C (HY-13418A) and rapamycin (HY-10219R) were from MCE (Monmouth, NJ, USA). Clodronate liposomes (F70101C-AC) were purchased from FormuMax Scientific (Sunnyvale, CA, USA). The primary antibodies against STING (#13647), phosphorylated TBK1 (p-TBK1, #5483), phosphorylated-IRF3 (p-IRF3, #4947), phosphorylated-p65 (p-p65, #3033), IκBα (#9242), phosphorylated-AMPKα (p-AMPKα, #2535), SQSTM1/p62 (#39749), LC3B (#43566), β-actin (#8457), and GAPDH (#2118) as well as the HRP-conjugated anti-rabbit (#7074) or anti-mouse (#7076) antibodies were purchased from Cell Signaling Technology (Boston, MA, USA). Human ELISA kits for TNF-α and IL-6 were purchased from Invitrogen (Grand Island, NY, USA). Cy5-PEG5000-SH was obtained from Ponsurebio (Shanghai, China). Live-Dead (L34962) fluorescence probe was purchased from Invitrogen (Carlsbad, CA, USA). Fluorochrome-labeled antibodies against mouse CD45 (#550994), CD11c (#562782), CD3 (#740113), CD19 (#557655), and Ly6G (#551460) were from BD Biosciences (New York, NJ, USA), whereas those against F4/80 (#123110) and CD11b (#101215) were from BioLegend (San Diego, CA, USA).

### Synthesis and physicochemical characterization of CSE-P12

P12 was made of a 13-nm gold nanoparticle (GNP) core decorated with hexapeptides on the surface. The GNP was synthesized as described in our previous study 20. P12 was prepared by mixing 1 volume of the hexapeptide (sequence of CLPFFD) solution (1 mM) with 10 volumes of the bare GNPs (~11 nM) for over 24 h incubation in the dark. The CSE was obtained from the combustion of commercially available cigarettes (10 mg tar and 0.8 mg nicotine, Marlboro Red, Phillip Morris, USA) according to our previously established method 19. Briefly, one cigarette was combusted, and the smoke was bubbled into two consecutive glass tubes (each contained 5 mL RPMI-1640 culture medium) using a modified syringe-driven apparatus (**Scheme 1A**). The collected CSE (considered as 100%) was filtered through a syringe filter (0.22 μm, Millipore, Billerica, MA, USA) and further diluted in the culture medium to a final concentration of 1% (volume percentage). The purified P12 was mixed with 1% CSE for 1 h prior to use.

The morphology and particle size of the bare GNPs, P12 and CSE-P12 were characterized by transmission electron microscopy (TEM) (HT7700, Hitachi, Tokyo, Japan) with an acceleration voltage of 80 kV. Their hydrodynamic sizes and zeta-potential were analyzed by a Zetasizer (Nano ZS, Malvern, Worcestershire, UK).

### Cell culture

The THP-1 cells (ATCC, RRID:CVCL_0006, Rockefeller, MD, USA) and THP-1 reporter cells (THP-1 XBlue, RRID: CVCL_X586, and THP-1 Blue ISG, RRID:CVCL_X588, InvivoGen, San Diego, CA, USA) were maintained in the complete RPMI 1640 medium containing 10% FBS (Vivacell, Shanghai, China), 2 mM L-glutamine and 1 mM sodium pyruvate (Grand Island, NY, USA) with 5% CO_2_ at 37 °C. The complete culture medium was supplemented with Zeocin at 200 or 100 μg/mL (InvivoGen, San Diego, CA, USA) as the selection medium for THP-1-XBlue cells or THP-1-ISG cells, respectively. The reporter cells were cultured with the selection medium every other passage. Cells were seeded into culture plates with the addition of PMA (50 ng/mL, Sigma, Sant-Louis, MO, USA) for 24 h to differentiate into macrophages, followed by PBS washing twice and resting for 48 h prior to further experiments.

### RNA-seq analysis

THP-1 cells were differentiated into macrophages and then stimulated with cGAMP (5 μg/mL) with or without P12 (100 nM), CSE and CSE-P12 (100 nM) treatment for 6 h; the total RNA was extracted using RNeasy mini kit (Qiagen, Hilden, Germany) and assessed for the purity by a Nanodrop Lite Spectrophotometer (Thermo Fisher Scientific, Waltham, MA, USA). The RNA-Seq was performed on the Illumina Novaseq 6000 platform with service provided by Novogene Co., Ltd. (Beijing, China).

The differentially expressed genes were determined by the DESeq2 package (version 1.38.3) in R (version 4.2.3). P.adjust < 0.0001 and |log2(fold change)| > 3 were defined as the thresholds for significant differential expression in the heat maps, whereas the thresholds were set to p.adjust < 0.05 and |log2(fold change)| > 1 for Venn diagrams. The gene list containing the log2FC (sorted in descending order) of all genes and their corresponding ENTREZID was used for GSEA by the clusterProfiler package (version 4.6.2). The pathways with |NES| > 1, NOM p-value < 0.05, and FDR (p.adjust) < 0.25 were regarded as significantly enriched. The significantly enriched top 20 up-regulated and top 20 down-regulated pathways for P12, CSE and CSE-P12 under cGAMP stimulation were presented.

### Reporter cell assay for the analysis of IRF and NF-κB/AP-1 activation

The THP-1 reporter cells were seeded (1×10^5^ cells/well) in a 96-well plate and differentiated into macrophages. They were stimulated with cGAMP (5 μg/mL) and treated with PBS, P12 (100 nM) or CSE-P12 (100 nM) for 24 h; the culture media were collected and centrifuged (14,000 rpm, 30 min, 4 °C) to remove the nanodevices. The supernatants (20 μL) were transferred into a new 96-well plate and mixed with the QUANTI-Blue solution (180 μL), which were incubated at 37 °C for 1-2 h until the color development. The color change was quantified by measuring the absorbance at 655 nm on a microplate reader (TECAN, Mannedorf, Zurich, Switzerland) to examine IRF or NF-κB/ AP-1 activation.

### Immunoblotting analysis

THP-1 cells (5×10^5^ cells/well) were seeded into a 24-well plate and differentiated into macrophages. Cells were stimulated with cGAMP (5 μg/mL) and treated with or without P12 (100 nM), CSE-P12 (100 nM), CSE (1%), or rapamycin (10 μM) for 4 h to assess STING expression and the downstream signaling. To examining autophagy induction, cells were pre-treated with autophagy inhibitor 3-MA for 1 h followed by cGAMP stimulation with or without CSE-P12 treatment for 4 h. To evaluate AMPK activation, cells were treated with P12, CSE, and CSE-P12 with or without cGAMP stimulation for 1 h; cells were pre-treated with the AMPK inhibitor compound C (5 μM) for 1 h, followed by cGAMP stimulation and CSE-P12 treatment for 1 h.

After various treatments, the cells were lysed with ice-cold RIPA buffer supplemented with Halt protease and phosphatase inhibitor cocktail (Beyotime, Shanghai, China). The total protein concentrations were measured using the Bradford assay kit (Thermo Fisher Scientific, Waltham, MA, USA), and adjusted to the same level. The proteins were separated by 10% SDS-PAGE and transferred to a PVDF membrane (Immobilon FL, Millipore, Billerica, MA, USA). The target proteins were probed by specific primary antibodies at 4 °C overnight, followed by incubation with HRP-conjugated secondary antibodies at room temperature for at least 1 h. The protein bands were imaged using the chemiluminescence method (ECL, Millipore, Billerica, MA, USA) on a ChemiDoc MP imaging system (Bio-Rad, Hercules, CA, USA). The protein band densitometry was analyzed using ImageJ software (NIH, Bethesda, MD, USA).

For tissue samples, weighted pieces of frozen mouse lungs were homogenized in the ice-cold RIPA buffer supplemented with Halt protease and phosphatase inhibitor cocktail. The homogenates were centrifuged (14,000 rpm, 4 °C, 10 min), and the supernatants were processed as described above for immunoblotting analysis.

### Cytokine analysis

THP-1 cells (5×10^5^ cells/well) were seeded and differentiated into macrophages in a 24-well plate. Cells were stimulated with cGAMP (5 μg/mL) and treated with or without P12 (100 nM), CSE or CSE-P12 (100 nM) for 24 h; the culture medium was collected and centrifuged (14,000 rpm, 4 °C, 30 min), and the supernatants were analyzed for the concentration of different cytokines (IL-6 and TNF-α) by ELISA following the manufacturer's instructions.

### Quantitative real-time PCR

Total RNA was extracted from cells or tissue samples by homogenization with TRIzol reagent (Gen star, Beijing China) and reverse-transcribed into cDNA using reverse transcriptase (Takara, Japan). The quantitative real-time PCR was performed using SYBR Green RT-PCR kits (Gen star, Beijing China). Relative mRNA expression was quantified using the 2^(-ΔΔCt)^ method. The primers used for real-time PCR assays were listed in the **Table S1**.

### Autophagy analysis

THP-1-Difluo™ hLC3 cells (from InvivoGen, San Diego, CA, USA) expressing the RFP-GFP-LC3B fusion protein were used to detect the autophagic flux. Cells were differentiated into macrophages, stimulated with cGAMP (5 μg/mL) and treated with or without P12 (100 nM) and CSE-P12 (100 nM) for different time periods (6, 12, and 24 h). After treatments, cells were washed 3 times with PBS and imaged on a confocal microscope (Olympus, Japan). During autophagosome formation, the GFP signals decreased progressively while the RFP signals increased. The numbers of RFP and GFP punctate dots were counted in each cell (> 45 cells for each sample), and averaged from three independent experiments.

### Temperature-dependent cellular uptake of CSE-P12 by TEM

Transmission electron microscopy (TEM) was employed to examine the cellular uptake and subcellular localization of CSE-P12. THP-1 cell-derived macrophages (5×10^6^ cells/well) in 6-well plates were incubated with CSE-P12 (100 nM) for 1 h at 37 °C or 4 °C. Cells were harvested and transferred to microcentrifuge tubes and fixed with 2.5% glutaraldehyde in PBS at room temperature for 30 min, and stored at 4 °C. The fixed samples were subsequently processed and imaged on a TEM by the Servicebio Co., Ltd. (Wuhan, China).

### ADP/ATP measurements by LC-MS/MS

The intracellular ATP and ADP concentrations were measured using liquid chromatography-tandem mass spectrometry (LC-MS/MS). Briefly, THP-1 cell-derived macrophages were first treated with P12 (100 nM) or CSE-P12 (100 nM) for 1 h. Cells were then washed with PBS, and collected in extraction buffer containing 80% methanol, which were sonicated by a probe ultrasonicator (Scientz, Ningbo, China) at 100 W for 2 min on ice to obtain the whole cell lysates. The lysates were then centrifuged at 12,000 rpm, 4 °C for 10 min, and the supernatants containing soluble metabolites were collected. The ATP and ADP levels were analyzed using an Orbitrap Exploris 480 Mass Spectrometer (Thermo Fisher Scientific, Waltham, MA, USA), and the data were processed using Xcalibur software (Thermo Fisher Scientific, Waltham, MA, USA).

### Preparation of viruses

WT-HAdV4 was produced at large scale in HEK293 cells, and purified by CsCl gradient ultracentrifugation. Virus titer was subsequently quantified by absorbance measurement at 260 nm. PR8 (A/Puerto Rico/8/34) was propagated in the chorioallantoic fluid of embryonated chicken eggs. For LD₅₀ determination, adult mice were challenged with serial virus dilutions through intranasal route.

### Viral pneumonia mouse models

C57BL/6J female mice (8 weeks, from Shanghai Jihui Biological Co., Ltd, Shanghai, China) or *Sting^-/-^* mice (kind gifts from Professor M. Du) were housed in the animal facility of Shanghai Public Health Clinical Center. Mice were infected with HAdV4 or PR8 to establish viral pneumonia mouse models. Mice were anesthetized with 2.5% 3-bromoethanol (Macklin, Shanghai, China) when needed. All mouse experiments in this study were approved by the Institutional Animal Care and Use Committee (IACUC) of Shanghai Public Health Clinical Center (Approval number: 2025-A011-01, 2025-A012-01) and conducted in a Biosafety Level-II Laboratory.

For HAdV4 infection, mice were given with HAdV4 virus (5×10^6^ PFU/per mouse) by intranasal administration. Mice were treated with PBS or CSE-P12 (500 nM, 50 μL) through intratracheal instillation 1 h before HAdV4 infection and on Day 2 and Day 4 after viral infection. Mice were sacrificed on Day 5 to collect lung tissues for further analysis.

For PR8 infection, mice were intranasally administered with PR8 virus (5500 PFU/per mouse). They were given with PBS or CSE-P12 (500 nM, 50 μL) through intratracheal instillation 1 h before viral infection, and on Days 2, 4, and 6 after viral infection. Mice were euthanized on Day 6 to harvest lung tissues for further analysis. In another set of experiments, mice were observed for 14 days to assess the survival rate.

To deplete pulmonary macrophages, clodronate liposomes (5 mg/mL, 75 μL) were administered via intratracheal injection 72 h before PBS or CSE-P12 (500 nM, 50 μL) treatment and 1 day after the HAdV4 infection (5×10^6^ PFU/per mouse); mice were treated with the same volume of empty liposomes as the control. CSE-P12 was given intratracheally 1 h before and on Day 2 and Day 4 after HAdV4 infection. On Day 5, the BALF and lung tissues were collected for further analysis.

### Viral loads in the mouse lung

The viral loads in the lung were measured via quantitative real-time PCR. The total RNA in the lung tissues was extracted for the detection of viral genes: Hexon gene for HAdV4 and M gene for PR8. The lung tissues were homogenized and resuspended in 1 mL of TRIzol reagent to extract total RNA. The mRNA levels for Hexon and M genes were quantified using a real-time PCR system. The primer pairs for Hexon and M genes were listed in the **Table S2**.

### CLP-induced sepsis mouse model

Wild-type C57BL/6J male mice (8 weeks, from SPF Biotechnology Co., Ltd, Beijing, China) or *Sting^-/-^* mice were used for the CLP-induced sepsis mouse model. All experimental procedures were approved by the Institutional Animal Care and Use Committee of Tianjin Medical University (TMUaMEC2020004).

CLP was performed according to the following procedures. Mice were operated for median abdominal incision under anesthesia with 3% sevoflurane by inhalation. The cecum was identified and ligated with nylon thread at the distal end. The ligated cecum was punctured once with a 21G needle to release a small amount of feces, and then placed back into the abdominal cavity. After the operation, mice were given saline (1 mL) subcutaneously, and were kept under observation until recovery from anesthesia. CSE-P12 (500 nM, 100 μL) was given through i.p. injection immediately after surgery and on Days 0, 1, 2, and 3 post-CLP. Mice were euthanized to collect the blood and major organs (e.g., lungs, spleen and kidneys) for further experiments. In another set of experiments, mice were observed for 7 days to analyze the survival rate.

### Bacterial counts

The tissue homogenates were prepared with pre-cooled aseptic PBS according to the ratio of 100 mg tissue pieces in 1 mL PBS, which were centrifuged at 2000 rpm, 4 °C for 10 min. The supernatants of the tissue homogenates and those of the whole blood were diluted and evenly spread on the LB agar dish, and cultured at 37 °C for 16-18 h. The CFU averaged to tissue weight (mg) was calculated to determine the severity of bacterial infection in major organs of CLP-induced sepsis mice.

### BALF collection and differential cell counting

The BALF was collected for total cell and differential cell counting. Briefly, at the end of the model, the mouse right hilum was clamped, and the ice-cold PBS (0.4 mL) was injected into the left lung lobe through the trachea twice; the BALF was collected and centrifuged at 1000 rpm, 4 °C for 10 min. The cell pallets were processed with red blood cell lysis buffer (Solarbio, Beijing, China) and resuspended in PBS for cell counting. Aliquots (50 μL) of the cell suspensions were cytospined (CytoSpin4, Thermo Fisher Scientific, Waltham, MA, USA) onto a glass slide, stained with Liu stain (Solarbio, Beijing, China), and imaged on an up-right microscope (ECLIPSE Ni-U, Nikon, Tokyo, Japan) for differential cell counting. At least 300 cells in total were counted for each sample.

### Lung histological analysis

For viral pneumonia mouse models, the right lower lobe or the left lung was processed for hematoxylin and eosin (H&E) staining. Pathological scores of the H&E stained images were assessed according to the following criteria: (i) no pathological changes, (ii) slightly perivascular infiltrates, (iii) perivascular and interstitial infiltrates less than 20% of the lobe sections, (iv) perivascular and interstitial infiltrates within the range of 20-50% of the lobe sections, (v) perivascular and interstitial infiltrates over 50% of the lobe sections.

For the CLP-induced sepsis mouse model, the left lung was processed for H&E staining. For each sample, H&E images at 400× magnification (≥ 20 images) were blindly scored by two independent researchers based on the five histopathological features: alveolar neutrophils, interstitial neutrophils, hyaline membranes, proteinaceous debris, and alveolar septal thickening.

### Flow cytometry analysis of CSE-P12 uptake in the lung immune cells

Cy5-labeled CSE-P12 (CSE-P12-Cy5) was intratracheally injected into HAdV4-infected mice, and the BALF and the lung tissues were collected after 24 h. The single cell suspensions from the BALF and the lung tissues were stained with viability dye and various fluorochrome-labeled antibodies to identify macrophages (CD45^+^CD11c^+^F4/80^+^), neutrophils (CD45^+^CD11b^+^Ly6G^+^), dendritic cells (CD45^+^CD11c^+^F4/80^-^), monocytes (CD45^+^CD11c^-^F4/80^+^), T cells (CD45^+^CD3^+^), and B cells (CD45^+^CD19^+^). The cells were first treated with anti-CD16/CD32 antibodies for 10 min to block the Fc receptors; they were then stained with a viability dye, fixed and incubated with a cocktail of different antibodies of interest for 30 min. The stained cells were analyzed on a LSRFortessa (BD, Franklin Lake, New Jersey, USA) flow cytometer, and the data were processed using FlowJo software (TreeStar, Ashland, OR, USA).

### Statistical analysis

All statistical tests were performed using GraphPad Prism 8.0. All data were presented as mean ± standard error of the mean (SEM), and p-value < 0.05 was considered statistically significant. For comparison between two groups, the student's t-test was used, whereas one-way ANOVA with Bonferroni post hoc test was performed for multiple comparisons among groups.

## Results

### Single-cell transcriptomics reveals elevated IFN responses in lung monocytes and macrophages under influenza infection

Monocytes and macrophages serve as the pivotal innate immune sentinels against pulmonary viral infections 21. To disclose their contribution to the over-activation of cGAS-STING signaling during viral infection, we analyzed the published single-cell RNA sequencing data from the influenza-infected mouse lungs 22. We observed distinct temporal shifts in myeloid populations: monocyte infiltration in the lung rose early at Day 3 post-infection, whereas macrophage numbers significantly increased by Day 9 (**Figure S1A**). To quantify the inflammatory states of these cells, the module scores for signatures associated with cGAS-STING activation were analyzed. The results showed that both monocytes and macrophages exhibited significantly elevated inflammatory scores in infected lungs, with the most dramatic increase occurring at Day 3 (**Figure S1B**). In addition, the type I interferon (IFN-α) response in monocytes was rapidly induced by Day 3 and sustained through Day 9, while macrophages showed a delayed IFN-α induction at Day 9 (**Figure S1B**). Consistent with these findings, the cGAS-STING downstream genes, the interferon-stimulated gene 15 (*Isg15*) (**Figure S1C**) and the C-X-C motif chemokine ligand 10 (*Cxcl10*) (**Figure S1D**), were significantly up-regulated in both cells. Collectively, these data suggested that modulating monocyte- and macrophage-specific pathogenic cGAS-STING signaling represents a potential therapeutic strategy for mitigating viral pneumonia.

### Fabrication and characterization of the drug-free nanodevice CSE-P12

**Figure 1:**
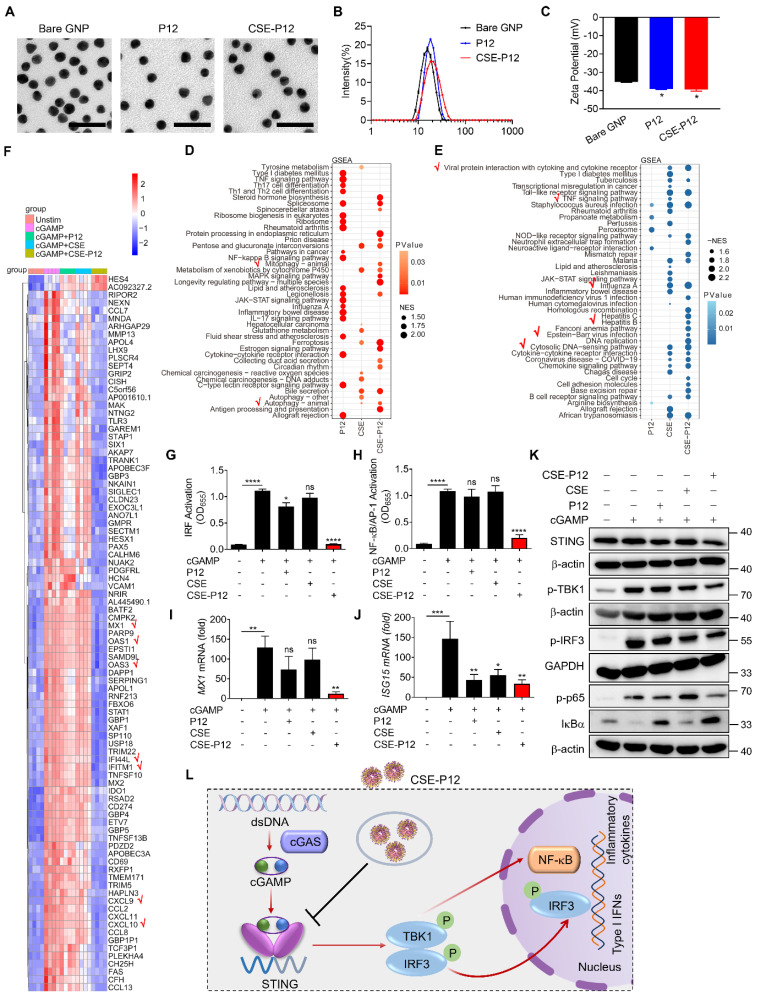
** The effects of CSE-P12 on the transcriptomic profile and cGAS-STING signaling pathway in THP-1 cell-derived macrophages under cGAMP stimulation.** (A-C) Physicochemical characterization of the bare GNPs, P12 and CSE-P12 by the TEM imaging for size and morphology (A), DLS analysis for the hydrodynamic sizes (B), and Zeta potential measurement for the surface charges (C); scale bar = 50 nm, N = 3. (D, E) The GSEA enrichment analysis of the differential expression genes (DEGs) for the top 20 up-regulated (D) and down-regulated (E) pathways for P12, CSE, and CSE-P12 treatments; the red check marks indicated the pathways of interest affected by CSE-P12; all pathways were significantly enriched with p < 0.05. (F) The heatmap showing top 91 DEGs of 5 experimental groups: Unstim, cGAMP, cGAMP+P12, cGAMP+CSE and cGAMP+CSE-P12; p < 0.0001 and log2 (fc) > 3 as the threshold; N = 4. (G, H) The effects of CSE-P12 on the activation of IRF (G) and NF-κB/AP-1 (H) upon cGAMP stimulation in the THP-1 reporter cell-derived macrophages; N = 3. (I, J) The effects of CSE-P12 on the mRNA levels of *MX1* (I) and *ISG15* (J) by RT-qPCR upon cGAMP stimulation for 24 h in THP-1-derived macrophages; N = 4. (K) Immunoblots showing the inhibitory effect of CSE-P12 on the STING expression and phosphorylation of TBK1 (p-TBK1), IRF3 (p-IRF3), and p65 (p-p65) as well as the degradation of IκBα in THP-1 cell-derived macrophages stimulated by cGAMP for 4 h; β-actin or GAPDH as the internal control; N = 3 for STING and p-TBK1, N = 4 for p-IRF3 and p-p65. (L) A scheme summarizing the inhibitory effect of CSE-P12 on the cGAS-STING pathway in macrophages. cGAMP = 5 μg/ mL, P12 = 100 nM, CSE = 1%, CSE-P12 = 100 nM. ns: not significant, *p < 0.05, **p < 0.01, ***p < 0.001, ****p < 0.0001 vs. cGAMP stimulation group unless otherwise indicated.

We previously developed a novel cigarette smoke extract (CSE)-modified peptide-gold nanoparticle (GNP) hybrid (CSE-P12) that induces protective autophagy for anti-inflammation 19. The CSE was freshly prepared as shown in **Scheme 1A**. The smoke extract was diluted to a final concentration of 1% when mixing with the peptide-GNP hybrid P12 to obtain CSE-P12. It was found that the CSE modification did not significantly affect the morphology of the nanodevice compared to the bare GNP and unmodified P12 from the TEM images (**Figure 1A**). The hydrodynamic sizes of CSE-P12 and P12 were determined to be 19.6 ± 0.03 nm and 18.0 ± 0.01 nm, respectively, which were 4.4 nm and 2.8 nm larger than that of the bare GNP (15.2 ± 0.04 nm) (**Figure 1B**). The Zeta potential of CSE-P12 (-39.4 ± 1.4 mV) was similar to that of P12 (-39.1 ± 0.5 mV); however, both were more negative than that of the bare GNP (-35.3 ± 0.4 mV) (**Figure 1C**). These results demonstrated the success in the synthesis of CSE-P12.

### CSE-P12 inhibited STING signaling pathway in THP-1 cell-derived macrophages

We previously discovered that CSE-P12 can induce protective autophagy to mitigate inflammatory responses in macrophages 19. Since autophagy could serve as a key mechanism for regulating STING activation 17, we hypothesized that CSE-P12 may attenuate the cGAS-STING signaling and its subsequent IFN-mediated inflammatory cascades. To define the regulatory role of CSE-P12 in this pathway, the high-throughput RNA-Seq analysis was conducted in THP-1 cell-derived macrophages under cGAMP stimulation. The Venn diagram demonstrated that cGAMP stimulation altered a total of 1704 gene expressions (1293 up-regulated; 411 down-regulated) (**Figure S2**). Notably, CSE-P12 inhibited 531 up-regulated genes by cGAMP, far exceeding the inhibitory effects of P12 (98 genes) or CSE (68 genes) alone (**Figure S2A**). Similarly, CSE-P12 increased 83 down-regulated genes, more than P12 (15 genes) or CSE (24 genes) alone (**Figure S2B**). This suggested that CSE-P12 exerts a significantly broader impact on the cGAMP-mediated transcriptome than its individual components P12 and CSE.

The GSEA pathway enrichment analysis further identified 18 up-regulated and 25 down-regulated pathways following the CSE-P12 treatment (**Figure 1D and E**). The up-regulated pathways were primarily associated with autophagy, ferroptosis, and protein processing in endoplasmic reticulum (**Figure 1D**). Differently, the down-regulated pathways were mainly enriched in immune signaling, specifically the cytosolic DNA sensing pathway, TNF signaling pathway, and viral infection-related signaling pathways (e.g., Influenza A and EBV infection) (**Figure 1E**). The heatmap analysis revealed that CSE-P12 suppressed the expression of several IFN-stimulated genes (ISGs), including *MX1*, *OAS1*, *OAS3*, *IFI44L*, *IFITM1*, *CXCL10*, and *CXCL9* (**Figure 1F**). These results confirmed the ability of CSE-P12 in modulating cGAS-STING signaling-associated biological processes.

It is known that cGAS-STING signaling activates TBK1 and the downstream transcription factors NF-κB and interferon regulatory factor 3 (IRF3). Using the reporter system for IRF and NF-κB/AP-1 activation, we observed that the CSE-P12 treatment dramatically reduced cGAMP-induced activation of both transcription factors in THP-1-cell-derived macrophages, while P12 or CSE alone showed negligible effects (**Figure 1G and H**). Notably, this inhibition was not due to the direct physical sequestration of cGAMP by CSE-P12; the supernatants from the cGAMP-CSE-P12 mixtures after centrifugation still maintained their ability to activate both IRF and NF-κB/AP-1, mirroring the activity of cGAMP alone (**Figure S3**).

We then confirmed that CSE-P12-mediated inhibition of IRF3 and NF-κB led to a functional reduction in effector molecules. CSE-P12 significantly inhibited the expression of ISGs (*MX1*,* ISG15* and* CXCL10*) (**Figure 1I and J, Figure S4A**) and the production of the pro-inflammatory cytokines IL-6 and TNF-α (**Figure S4B and C**). Moreover, the immunoblotting analysis showed that CSE-P12 decreased the total STING protein levels, down-regulated cGAMP-induced phosphorylation of TBK1, IRF3, and p65, and inhibited the degradation of IκBα (**Figure 1K, Figure S5**). Taken together, these results demonstrated that CSE-P12 attenuated the expression of ISGs and pro-inflammatory cytokines by specifically inhibiting the STING-mediated IRF3 and NF-κB activation in macrophages (**Figure 1L**).

### CSE-P12 promoted autophagy-mediated STING degradation to down-regulate cGAS-STING signaling

Studies have shown that STING expression is regulated by autophagy-dependent degradation 17. Our transcriptomic analysis indicated that CSE-P12 significantly up-regulated genes associated with autophagy process (**Figure 1D**). Furthermore, the immunoblots demonstrated that CSE-P12 reduced STING protein levels and suppressed downstream signaling following cGAMP stimulation (**Figure 1K**). Based on these observations, we hypothesized that CSE-P12 facilitates STING degradation by inducing autophagy, thereby down-regulating the cGAS-STING pathway.

**Figure 2:**
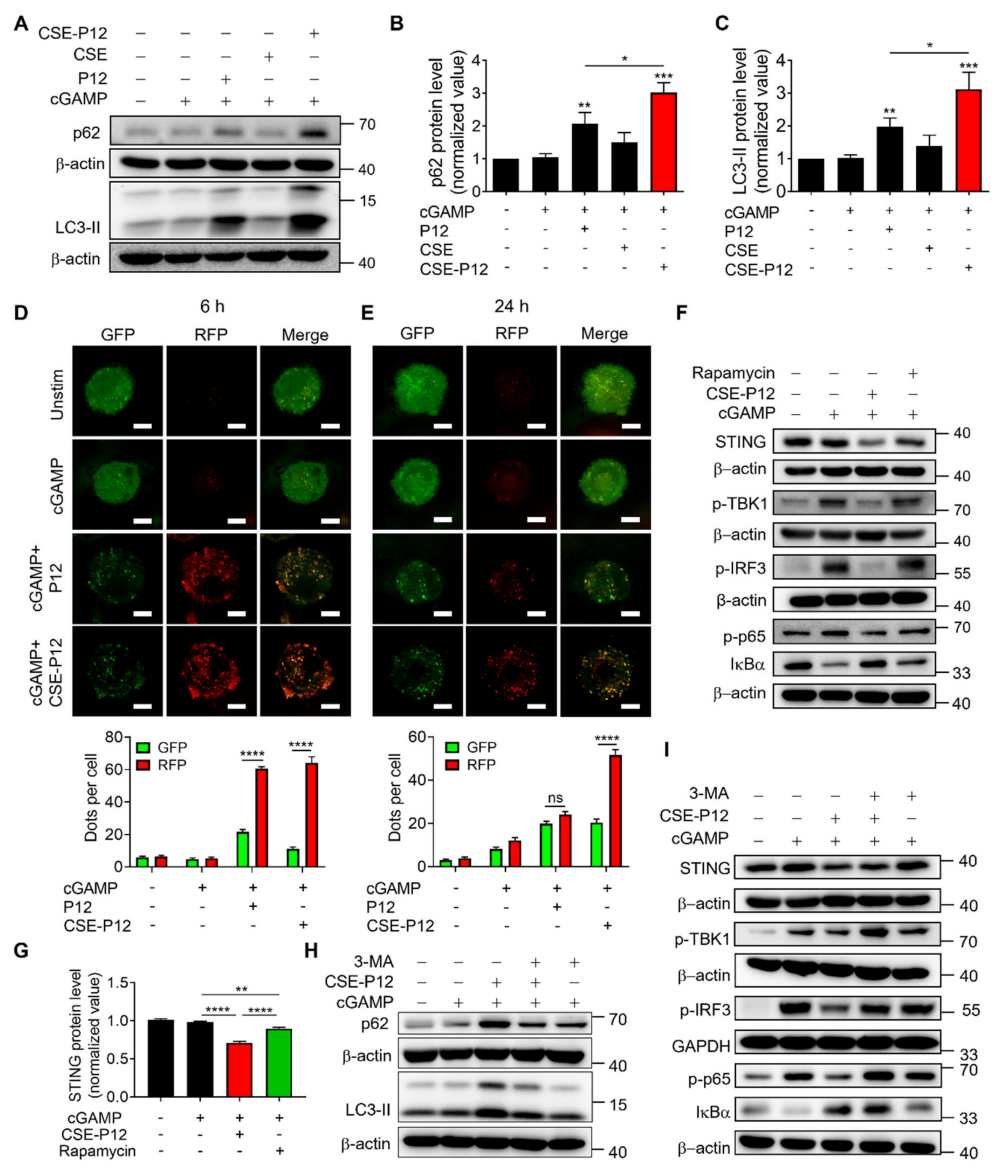
** Autophagy induction by CSE-P12 for STING degradation inhibiting cGAS-STING signaling in THP-1 cell-derived macrophages.** (A) Immunoblots showing the effect of CSE-P12 on p62 and LC3-II protein expression upon cGAMP stimulation for 4 h; β-actin as the internal control. (B, C) The densitometry analysis of p62 (B) and LC3-II (C) levels in (A); N = 5 for p62, N = 4 for LC3-II; P12 and CSE-P12 = 100 nM, cGAMP = 5 μg/mL; *p < 0.05, **p < 0.01, ***p < 0.001 vs. the cGAMP stimulation group unless otherwise specified. (D, E) Representative confocal images of the RFP-GFP-LC3 fusion protein (top) and quantitative analysis of RFP and GFP dots (bottom) in the THP-1 LC3 reporter cell-derived macrophages treated with P12 or CSE-P12 under cGAMP stimulation for 6 h (D) and 24 h (E); scale bar = 5 μm; more than 45 cells in each group were quantified; P12 and CSE-P12 = 100 nM, cGAMP = 5 μg/mL; N = 3. (F) Immunoblots showing the effects of CSE-P12 and Rapamycin on the levels of STING, p-TBK1, p-IRF3, p-p65, and IκBα in THP-1 cell-derived macrophages under cGAMP stimulation for 4 h; β-actin or GAPDH as the internal control; CSE-P12 = 100 nM, rapamycin =10 μM, cGAMP = 5 μg/mL. (G) The densitometry analysis of STING levels in (F); N = 3. (H) The immunoblots showing the effects of the pretreatment of the autophagy inhibitor 3-MA (10 μM, 1 h) on the protein levels of p62 and LC3-II in THP-1 cell-derived macrophages under cGAMP stimulation for 4 h with/without CSE-P12 treatment; β-actin as the internal control. (I) The immunoblots showing the effects of the pretreatment of the autophagy inhibitor 3-MA (10 μM) for 1 h on the levels of STING, p-TBK1, p-IRF3, p-p65 and IκBα in THP-1 cell-derived macrophages under cGAMP stimulation for 4 h with or without CSE-P12 treatment; β-actin or GAPDH as the internal control; CSE-P12 = 100 nM, cGAMP = 5 μg/mL. ns: not significant, **p < 0.01, ****p < 0.0001.

To test this hypothesis, we first examined the effect of CSE-P12 on the autophagic markers p62 and LC3-II. It was found that both P12 and CSE-P12 elevated the levels of p62 and LC3-II under cGAMP stimulation; however, CSE-P12 exhibited greater autophagy induction ability than P12 (**Figure 2A-C**). We then monitored the autophagic flux using a THP-1-Difluo hLC3 reporter cell system, which utilizes the microtubule associated protein 1 light chain 3 beta (LC3B) protein fused with an acid-sensitive green fluorescent protein (GFP) and an acid-stable red fluorescent protein (RFP). In this system, the formation of the acidic autolysosomes quenches the GFP signal while increasing in the RFP signal upon autophagy induction. Compared to the P12-treated cells, the CSE-P12 treatment under cGAMP stimulation led to a sustained increase in punctate RFP signals (the formation of autophagosomes) and a concomitant loss of GFP signals (the fusion of autophagosomes with lysosomes) (**Figure 2D and E**). Notably, while P12-induced autophagic flux peaked at 6 h and declined by 24 h, CSE-P12 maintained strong induction throughout the 24 h period; cGAMP stimulation alone had no effect on autophagy. These results confirmed that CSE-P12 was capable of promoting cGAMP-induced autophagic process in macrophages.

Next, we evaluated the functional impact of CSE-P12-induced autophagy on cGAS-STING signaling. It was found that CSE-P12 was more potent than the classical autophagy activator rapamycin in facilitating STING degradation (**Figure 2F and G**); this finding was corroborated by the autophagic flux assays (**Figure S6**). While rapamycin only modestly decreased cGAMP-induced TBK1 phosphorylation, CSE-P12 significantly inhibited the phosphorylation of TBK1, IRF3, and p65, as well as the degradation of IκBα (**Figure 2F**). This inhibition effectively down-regulated *IFNB1* expression downstream of the cGAS-STING pathway (**Figure S7**). Importantly, these effects of CSE-P12 were reversible: the autophagy inhibitor 3-methyladenine (3-MA) not only blocked CSE-P12-induced autophagy (**Figure 2H**), but also rescued STING levels and restored TBK1 and IRF3 phosphorylation as well as NF-κB activation (**Figure 2I, Figure S8**). Collectively, these results demonstrated that CSE-P12 suppressed STING-mediated type-I IFN and NF-κB signaling by promoting autophagic degradation of STING.

### CSE-P12 ameliorated lung inflammation and injuries in the HAdV4-induced pneumonia mouse model

**Figure 3:**
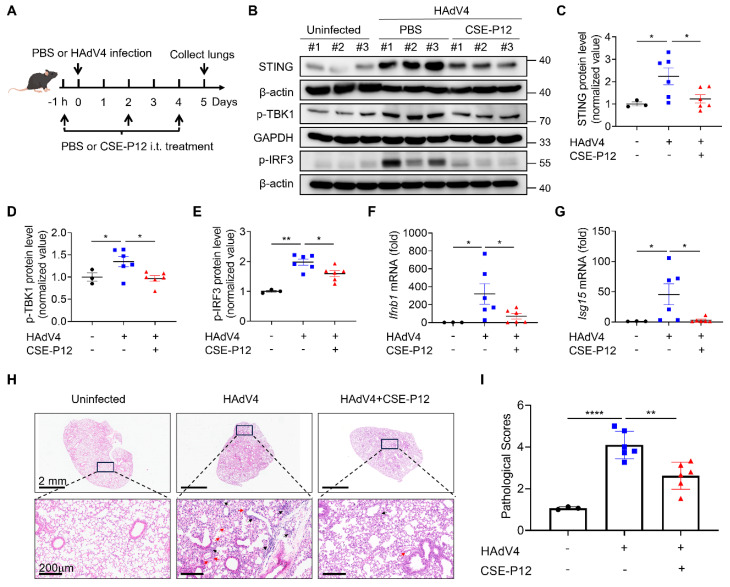
** The protective effects of the drug-free nanodevice CSE-P12 on DNA virus HAdV4-induced lung injury in mice.** (A) The viral pneumonia mouse model by HAdV4 infection through intranasal administration (5×10^6^ PFU per mouse); mice were treated with PBS or CSE-P12 (500 nM, 50 μL) through intratracheal (i.t.) injection at 1 h before and on Day 2 and Day 4 after infection. (B) Immunoblots showing the expression of STING and phosphorylated TBK1 (p-TBK1) and IRF3 (p-IRF3) in the cGAS-STING pathway in the lung tissues; β-actin or GAPDH as the internal control. (C-E) The densitometry analysis on the expression of STING (C), p-TBK1 (D), and p-IRF3 (E) in the immunoblots in (B). (F, G) The mRNA levels of *Ifnb1* (F) and *Isg15* (G) in the mouse lung on Day 5 by RT-qPCR. (H) The H&E images of mouse lung sections from uninfected (PBS), HAdV4 infected, and HAdV4+CSE-P12 groups; scale bar = 2 mm (top) or 200 μm (bottom); black arrows indicated the perivascular and interstitial infiltration of inflammatory cells and the lung consolidation; red arrows indicated infiltrated neutrophils. (I) Histological scores of the H&E images in (H); N = 3 for the PBS group, N = 6 for the HAdV4 and HAdV4+CSE-P12 groups. *p < 0.05, **p < 0.01, ***p < 0.001, ****p < 0.0001.

Viral pneumonia is a severe inflammatory complication of the lung often precipitated by respiratory infections from DNA or RNA viruses, such as influenza and SARS-CoV-2 23. Given that the cGAS-STING signaling pathway is the primary driver of the host immune responses to viral DNA 24, we investigated whether CSE-P12 could modulate this pathway to mitigate lung inflammation in vivo. For such purpose, we established a viral pneumonia mouse model by intranasal inoculation of human adenovirus type 4 (HAdV4) with or without the CSE-P12 treatment via intratracheal injection at -1 h, Day 2, and Day 4 post-infection (**Figure 3A**). It was found that the CSE-P12 treatment remarkably attenuated the HAdV-4-induced elevation of STING protein expression, as well as the phosphorylation of TBK1 (p-TBK1) and IRF3 (p-IRF3) in the lung tissues (**Figure 3B-E**). Consistent with this inhibition, the expression of the downstream pro-inflammatory genes *Ifnb1* and *Isg15* was significantly reduced on Day 5 (**Figure 3F and G**). These results indicated that CSE-P12 effectively suppressed HAdV4-triggered cGAS-STING activation in the lung.

Histopathological assessment via H&E staining further confirmed the protective effects of CSE-P12. The CSE-P12 treatment significantly decreased perivascular and interstitial infiltration of inflammatory cells when compared with the HAdV4-infected group (**Figure 3H and I**). Notably, the body weight kinetics or the HAdV4 viral load on Day 5 were not altered by CSE-P12 (**Figure S9**). This suggests that CSE-P12 effectively suppresses harmful, virus-mediated hyper-inflammation without compromising the host primary antiviral immunity—a critical distinction for therapeutic safety.

### The protective effects of CSE-P12 on HAdV4-induced pneumonia were diminished in *Sting*^-/-^ mice

**Figure 4:**
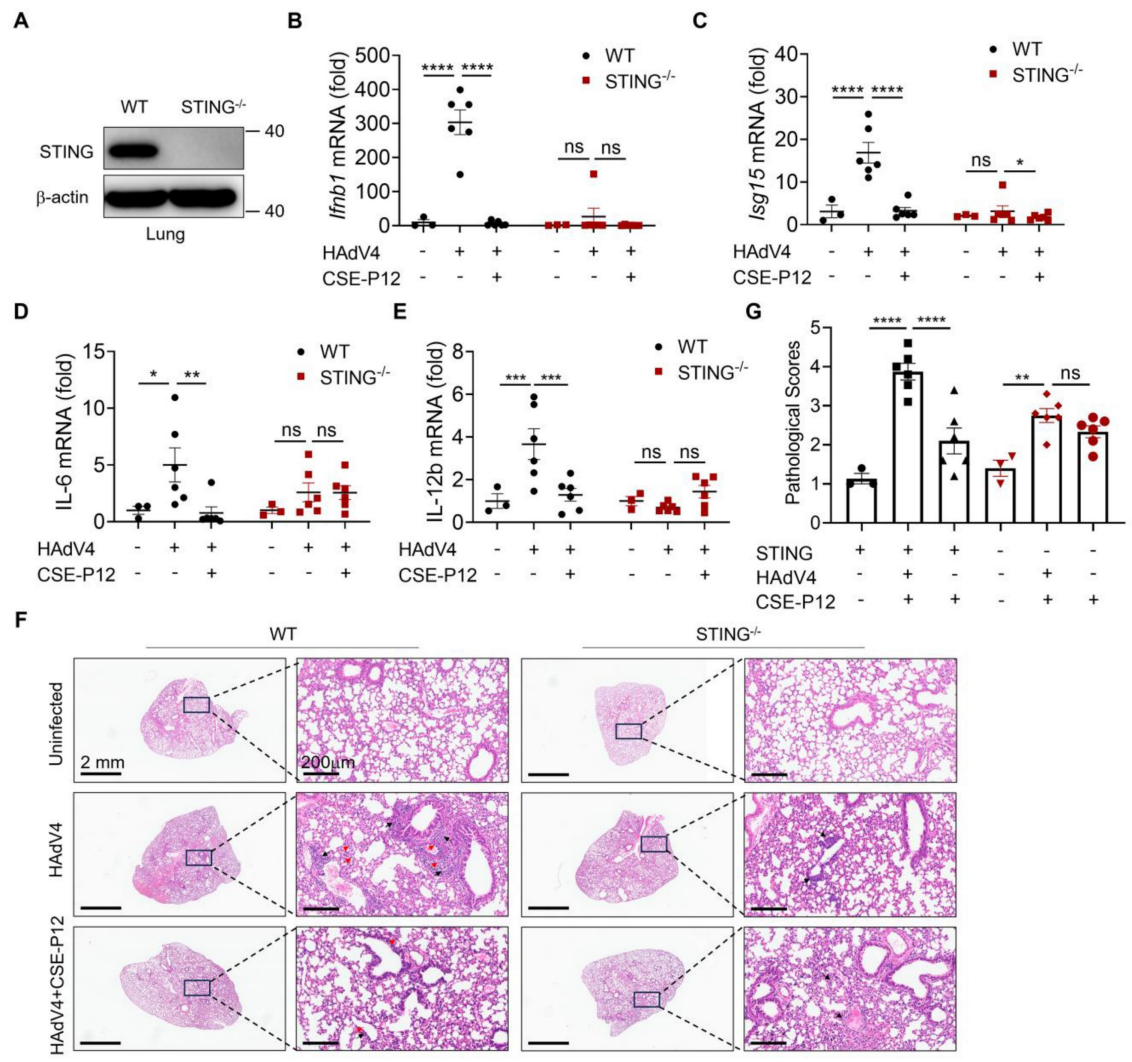
** CSE-P12 alleviated HAdV4-induced lung injury through inhibiting the STING pathway.** (A) Immunoblots showing the absence of STING in the lung of STING^-/-^ mice; β-actin as the internal control. (B, C) The effects of CSE-P12 on the mRNA levels of *Ifnb1* and *Isg15* genes in the lung of WT and STING^-/-^ mice on Day 5 after HAdV4 infection. (D, E) The effects of CSE-P12 on the mRNA levels of IL-6 (D) and IL-12b (E) in the lung of WT and STING^-/-^ mice on Day 5 after HAdV4 infection; CSE-P12 = 500 nM (50 μL), 3 doses at -1 h, 48 h, and 96 h after infection. (F) The representative H&E images of lung sections from WT and STING^-/-^ mice in each group; scale bar = 2 mm (left) and 200 μm (right); black arrows indicated the perivascular and interstitial infiltration of inflammatory cells and the lung consolidation; red arrows indicated infiltrated neutrophils in the lung. (G) Histological scores quantified from the H&E images in (F). N = 3 for the uninfected group, N = 6 for HAdV4 infected and HAdV4+CSE-P12 groups; ns: not significant, *p < 0.05, **p < 0.01, ***p < 0.001, ****p < 0.0001.

To investigate whether CSE-P12 ameliorated viral pneumonia through regulating the cGAS-STING signaling pathway, we evaluated its therapeutic effects in *Sting^-/-^* mice under HAdV4 infection. The absence of STING protein in the lung of *Sting^-/-^* mice was confirmed by immunoblotting (**Figure 4A**). Upon HAdV4 infection, the elevation of *Ifnb1* and *Isg15* mRNA levels was significantly lower in *Sting^-/-^* mice when compared to WT mice (**Figure 4B and C**), confirming the impairment of the cGAS-STING pathway. Similarly, the CSE-P12 treatment significantly decrease the expression of the proinflammatory cytokines IL-6 and IL-12b in WT mice, but not in *Sting*^-/-^ mice (**Figure 4D and E**). The histological analysis of the lung tissues further demonstrated that CSE-P12 reduced the pathological scores in HAdV4-infected WT mice, but failed to provide protection in *Sting*^-/-^ mice (**Figure 4F and G**). Collectively, these results indicated that CSE-P12 exerted the therapeutic effects on HAdV4-induced pneumonia through suppressing cGAS-STING signaling.

### CSE-P12 targeted pulmonary macrophages to intervene virus-induced lung inflammation and injury

**Figure 5:**
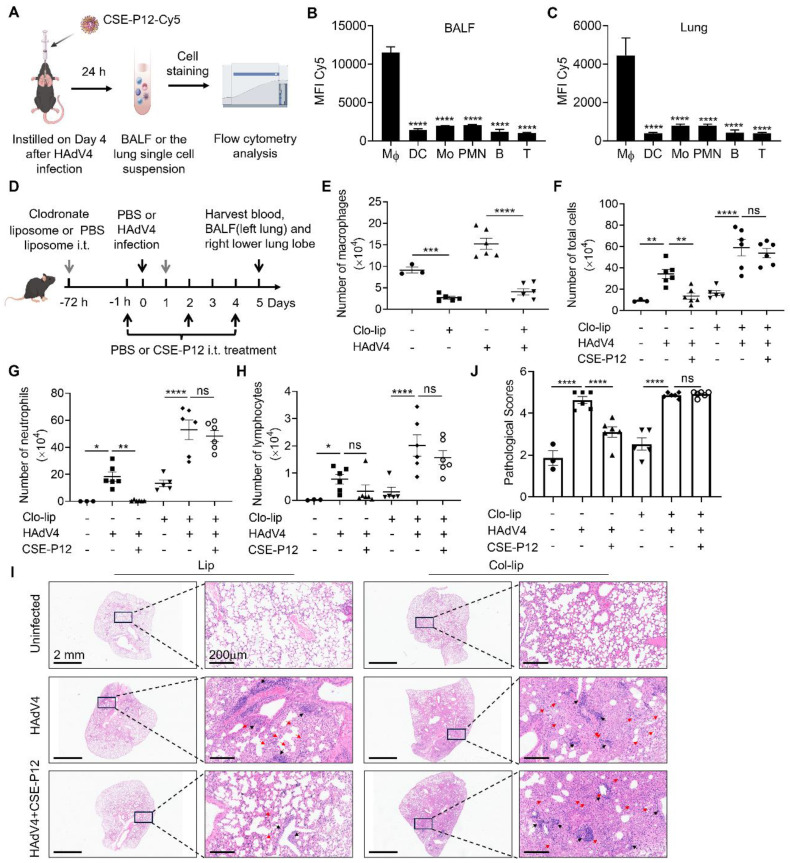
** The macrophage specific inhibition by the intratracheally administered CSE-P12 protected mice from HAdV4-induced lung inflammation and injury.** (A) Schematic illustration showing the process of flow cytometry analysis on the accumulation of Cy5-labeled CSE-P12 in the immune cells from the BALF and lung tissues of HAdV4 infected mice; CSE-P12-Cy5 was given intratracheally on Day 4 after viral infection. (B, C) MFI analysis of the CSE-P12-Cy5 internalized by different immune cells in the BALF (B) and in the lung tissues (C); Mφ: macrophages, DC: dendritic cells, Mo: monocytes, PMN: neutrophils; B: B cells, T: T cells; N = 5; ****p < 0.0001, vs. macrophages. (D) A scheme showing the HAdV4 infection (5×10^6^ PFU per mouse) mouse model with macrophage depletion in the lung by clodronate liposomes (75 μL, 5 mg/mL) through i.t. injection 72 h before and 1 day after infection; CSE-P12 (50 μL, 500 nM) was given intratracheally 1 h before the viral challenge; PBS was given as the uninfected and untreated controls. (E-H) The BALF was collected on Day 5 to assess the efficiency of macrophage depletion (without CSE-P12 treatment) (E), and to analyze the effects of CSE-P12 on the number of total cells (F), neutrophils (G) and lymphocytes (H). (I) The representative H&E images of the lung sections from each group; scale bar = 2 mm (left) and 200 μm (right); black arrows indicated the perivascular and interstitial infiltration of inflammatory cells and the lung consolidation; red arrows indicated infiltrated neutrophils. (J) The histological scores quantified from the H&E images in (I). Lip: empty liposomes, Clo-lip: clodronate liposomes. ns: not significant, *p < 0.05, **p < 0.01, ***p < 0.001, ****p < 0.0001.

To identify the primary cellular targets of CSE-P12 in the lung, the Cy5-labeled CSE-P12 (CSE-P12-Cy5) was administered intratracheally to track its uptake across various immune cell populations in the bronchoalveolar lavage fluid (BALF) and lung parenchyma of the HAdV4-infected mice (**Figure 5A**). The different immune cell subsets were identified by flow cytometry, including macrophages (F4/80^+^CD11c^+^), dendritic cells (F4/80^-^CD11c^+^), monocytes (F4/80^+^CD11c^-^), neutrophils (Ly6G^+^CD11b^+^), T cells (CD3^+^), and B cells (CD19^+^) (**Figure S10A**). By comparing the mean fluorescence intensity (MFI) of Cy5 across these populations, we found that CSE-P12-Cy5 was predominantly internalized by macrophages (**Figure 5B and C, Figure S10B**). These results indicated that pulmonary macrophages were the primary targets of CSE-P12 following intratracheal instillation.

Next, to verify whether these macrophages served as the essential effector cells for the observed therapeutic benefits of CSE-P12, they were depleted by intratracheal administration of the clodronate liposomes in the HAdV4 mouse model. Clodronate liposomes were given 72 h prior to the CSE-P12 treatment and 24 h post-infection to ensure sustained depletion; CSE-P12 was subsequently administered 1 h before infection and on Days 2 and 4 post-infection (**Figure 5D**). By Day 5, the clodronate treatment had achieved ~70% reduction of macrophages in the BALF across both uninfected (PBS) and HAdV4-infected groups (**Figure 5E**). In addition, the ability of CSE-P12 to reduce total cell infiltration (**Figure 5F**) and neutrophil recruitment (**Figure 5G**) was abrogated in macrophage-depleted mice, whereas the protective effects of CSE-P12 remained robust in the control liposome group. Interestingly, the CSE-P12 intervention did not alter lymphocyte infiltration regardless of the macrophage status (**Figure 5H**).

Histopathological analysis on the H&E images further confirmed these findings. In the absence of macrophages, CSE-P12 failed to reduce lung perivascular and interstitial infiltration of inflammatory cells or improve overall pathological scores (**Figure 5I and J**). These results demonstrated that pulmonary macrophages were indispensable for the CSE-P12-mediated mitigation of HAdV4-induced pneumonia. Furthermore, we observed that the efficacy of CSE-P12 extended beyond DNA viruses, as it also significantly ameliorated lung inflammation and injury induced by the RNA virus PR8 (**Figure S11**).

### The protective effects of CSE-P12 in the CLP-induced sepsis mouse model

**Figure 6:**
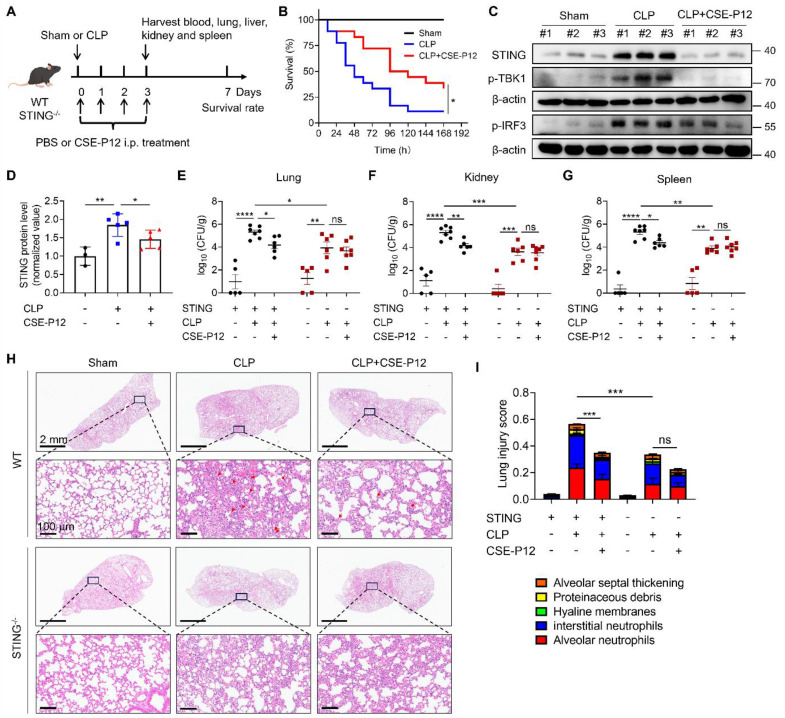
** CSE-P12 ameliorated the survival rate and lung inflammation in the CLP-induced sepsis mouse model by regulating STING pathway.** (A) A scheme showing the CLP-induced sepsis mouse model in WT and STING^-/-^ mice; CSE-P12 treatment (500 nM, 100 μL) or the PBS control was intraperitoneally administered on Day 0, 1, 2 and 3, and mice were monitored for survival rate over 7 days or sacrificed on Day 3 for further analysis. (B) The survival rate of mice over 7 days after CLP with/without CSE-P12 treatment; N = 6 for the sham group and N = 18 for the CLP and CSE-P12 treatment groups; the log-rank (Mantel-Cox) test was used for the statistical analysis. (C) Immunoblots showing the levels of STING, p-TBK1, and p-IRF3 in the lung tissues of mice from the sham control, CLP, CLP+CSE-P12 groups; β-actin as the internal control. (D) Densitometry analysis of STING protein levels in (C). (E-G) Bacterial loads in the lung (E), kidneys (F), and spleen (G) of WT and STING^-/-^ mice under CLP-induced sepsis with CSE-P12 (500 nM, 100 μL) or PBS treatment through i.p. injection (Day 3 after CLP). (H) The representative H&E images of lung sections from WT and STING^-/-^ mice under CLP with/without CSE-P12 treatment (Day 3); scale bar = 2 mm (top) and 100 μm (bottom). The red arrows indicate neutrophils in the lung. (I) The lung injury scores were analyzed based on the 5 pathophysiological features in the H&E images in (H); N = 3 for sham control and N = 5 for CLP and treatment groups. ns: not significant, *p < 0.05, **p < 0.01, ***p < 0.001, ****p < 0.0001.

Sepsis is characterized by a systemic inflammatory response to pathogenic infection, often leading to multi-organ dysfunction 25. As the aberrant cGAS-STING activation is known to exacerbate sepsis-associated inflammation 26, we evaluated the therapeutic potential of CSE-P12 in a polymicrobial sepsis mouse model induced by cecal ligation and puncture (CLP) (**Figure 6A**).

The CSE-P12 treatment was given by intraperitoneal injection immediately after the CLP procedure and at 24, 48, and 72 h post-operation. The treatment significantly increased the 7-day survival rate (**Figure 6B**) without affecting the mouse body weight (**Figure S12A**). Notably, the CSE-P12 treatment reduced the bacterial loads in the lung, kidneys, and spleen (**Figure S12B-D**). At the molecular level, CSE-P12-mediated inhibition of the cGAS-STING pathway in the lung (Day 3) was evidenced by the decrease in the levels of the STING protein and phosphorylated TBK1 and IRF3 (**Figure 6C and D, Figure S13**). The histopathological assessment on the H&E images also confirmed the protective effects of CSE-P12 on CLP-induced lung injury (**Figure S14A-E**). Similarly, CSE-P12 effectively inhibited CLP-induced glomerular atrophy and tubular dilation in the kidney (**Figure S14F**), and prevented the expansion and fusion of the white pulp and inflammatory cell infiltration in the spleen (**Figure S14G**).

To verify the importance of STING in the observed protective effects of CSE-P12, *Sting*^-/-^ mice were employed for the same CLP model in **Figure 6A**. Consistent with previous studies that STING deficiency decreases bacterial burden in the intestine of CLP-induced sepsis mice 27, we observed that *Sting*^-/-^ mice exhibited lower bacterial loads in the lung, kidneys, and spleen when compared with the WT mice (**Figure 6E-G**). More importantly, the ability of CSE-P12 to reduce bacterial loads (**Figure 6E-G**) and attenuate lung injury, including the overall lung injury score, alveolar neutrophil, and alveolar septal thickening (**Figure 6H and I, Figure S15**), were abrogated in *Sting*^-/-^ mice. These results confirmed that STING was essential for driving lung inflammation in CLP-induced sepsis, and that CSE-P12 exerted the therapeutic effects by specifically targeting the cGAS-STING pathway.

### CSE-P12 induced autophagy through energy-dependent AMPK activation

**Figure 7:**
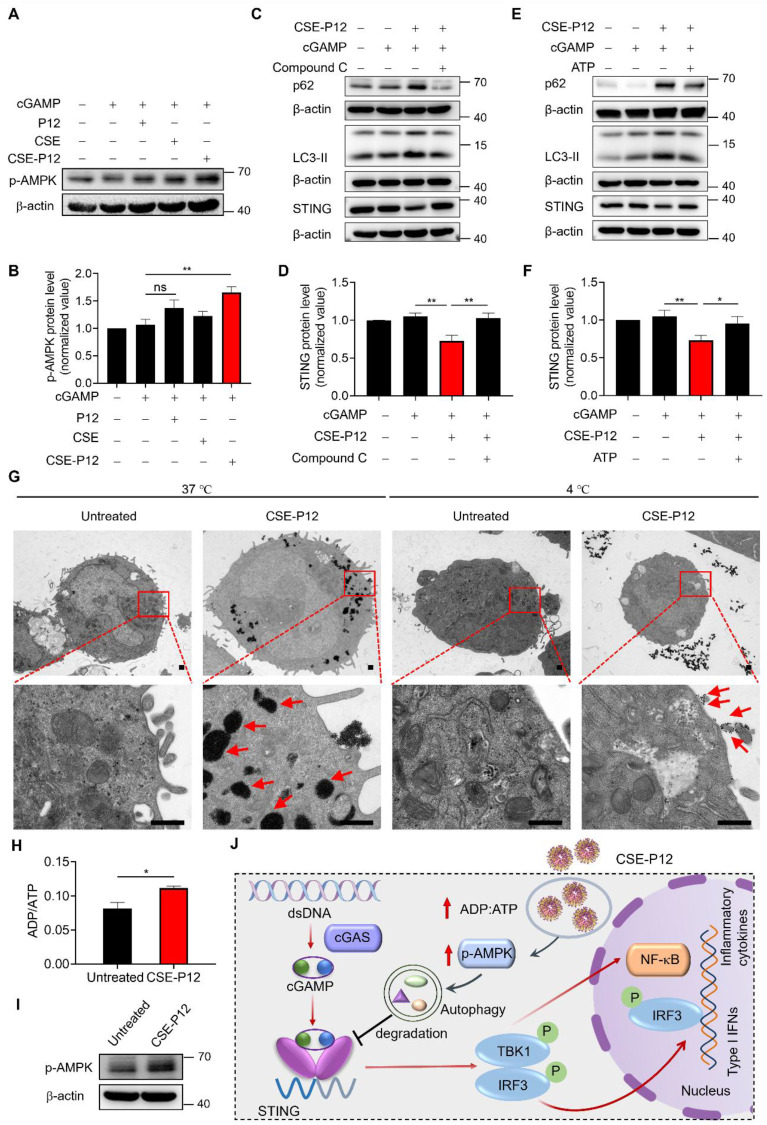
** CSE-P12 facilitated autophagy-mediated STING degradation by promoting energy-dependent AMPK activation.** (A) Immunoblots showing the effect of CSE-P12 on the phosphorylation of AMPK (p-AMPK) in THP-1 cell derived macrophages under cGAMP stimulation for 1 h; β-actin as the internal control; cGAMP = 5 μg/mL, P12 = 100 nM, CSE = 1%, CSE-P12 = 100 nM. (B) Densitometry analysis of p-AMPK levels in (A); N = 4. (C) Immunoblots showing the effects of the pretreatment (1 h) of the AMPK inhibitor Compound C (5 μM) on the protein levels of p62, LC3-II, and STING in THP-1 cell-derived macrophages under cGAMP stimulation for 4 h with or without CSE-P12 co-treatment; β-actin as the internal control; cGAMP = 5 μg/mL, CSE-P12 = 100 nM. (D) Densitometry analysis of STING levels in (C); N = 3. (E) Immunoblots showing the effects of the addition of ATP (10 μM) on the protein levels of p62, LC3-II, and STING in THP-1 cell-derived macrophages under cGAMP stimulation for 4 h with or without CSE-P12 co-treatment; β-actin as the internal control; cGAMP = 5 μg/mL, CSE-P12 = 100 nM. (F) Densitometry analysis of STING levels in (E); N = 4. (G) Representative TEM images showing the uptake of CSE-P12 in THP-1 cell-derived macrophages at 37 ℃ or 4 ℃; red arrows indicated nanoparticle clusters; CSE-P12 = 100 nM for 1 h; the scale bar = 500 nm. (H) ADP-to-ATP ratios measured by LC-MS in THP-1 cell-derived macrophages with or without CSE-P12 treatment; N = 3. (I) Immunoblots showing the effect of CSE-P12 treatment (1 h) on p-AMPK; β-actin as the internal control. (J) A scheme summarizing the inhibitory mechanism of CSE-P12 on cGAS-STING pathway through energy-dependent induction of autophagy in macrophages. ns: not significant, *p < 0.05, **p < 0.01, ***p < 0.001.

To elucidate the mechanism by which CSE-P12 facilitated autophagy, we examined two known regulatory pathways: 5' AMP-activated protein kinase (AMPK) and Akt-mTOR axis 28, 29. While mTORC1 suppresses the biogenesis of autophagosomes 30, AMPK activation triggers autophagy 28. The immunoblotting results showed that the phosphorylated mTOR (p-mTOR) levels remained unchanged following the CSE-P12 treatment in cGAMP-stimulated THP-1 cell-derived macrophages, despite an observed elevation in phosphorylated Akt (p-Akt) (**Figure S16**). This suggested that the Akt-mTOR pathway may not be the key driver for autophagy induction by CSE-P12. In contrast, CSE-P12 significantly increased AMPK phosphorylation (p-AMPK) under cGAMP stimulation (**Figure 7A and B**). This elevation was reversed by the AMPK inhibitor compound C (**Figure 7C and D, Figure S17 A and B**) or by the addition of extracellular ATP (**Figure 7E and F, Figure S17 C and D**). Collectively, these findings confirmed that AMPK activation was essential for CSE-P12-mediated autophagy and subsequent STING degradation.

As AMPK is a highly conserved sensor of cellular energy fluctuations, it is typically activated by an increased ADP/ATP ratio (i.e., energy exhaustion). The TEM images demonstrated that the cellular uptake of CSE-P12 was energy-dependent, where a near-total absence of internalization was observed at 4 °C compared to 37 °C (**Figure 7G**). Thus, we hypothesized that the rapid, large-scale internalization of CSE-P12 depleted cellular energy reserves, triggering the AMPK stress response for autophagy induction. This hypothesis was supported by the LC-MS results, where the intracellular ADP/ATP ratio was significant increased within 1 h of CSE-P12 treatment (**Figure 7H**), correlating with elevated p-AMPK (**Figure 7I**). These data revealed that the high energy demand of CSE-P12 uptake shifted the metabolic state of macrophages, activating the AMPK-autophagy axis to facilitate STING degradation and inhibition of cGAS-STING signaling (**Figure 7J**).

## Discussion

The cGAS-STING sensing machinery is a fundamental component of the innate immune system, essential for host defense against a broad spectrum of viral and bacterial pathogens 31. However, cGAS-STING signaling is a “double-edged sword”; while necessary for initial defense, its dysregulated or chronic activation drives a variety of life-threatening inflammatory diseases 32. Current therapeutic strategies to control cGAS-STING-mediated pathogenic inflammation largely rely on systemic immunosuppressive drugs, such as corticosteroids, which lack specificity and are associated with undesired side effects 33. Despite the clear clinical need, specific STING inhibitors have yet to be approved for clinical uses. In this study, we identified CSE-P12 (hexapeptide-coated GNPs with a tiny amount of CSE modification) as a potent immunomodulator capable of attenuating cGAS-STING-induced IFN responses and harmful inflammation in macrophages. Mechanistically, CSE-P12 promoted the protective autophagy for STING degradation. This was driven by the rapid, high-capacity internalization of the nanodevices, which imposed a localized metabolic demand that activated the energy-sensing AMPK pathway. In addition, CSE-P12 exhibited an efficient capability of targeting lung macrophages, enabling the effective down-regulation of excessive STING activation in various pathological contexts, including HAdV4 (DNA virus) and PR8 (RNA virus)-induced pneumonia, as well as polymicrobial sepsis. These findings suggest that CSE-P12 represents a promising therapeutic approach for mitigating cGAS-STING-driven inflammatory organ injury.

### Precision modulation of cGAS-STING signaling in macrophages emerges as a therapeutic strategy for treating infectious inflammatory lung diseases

The cGAS-STING axis serves as a “signaling rheostat”, finely tuning the balance between protective host defense and pathological inflammatory responses. While this pathway is indispensable for combatting diverse pathogens including DNA/RNA viruses and bacteria 34, multiple lines of evidence highlight its “double-edged” action in pulmonary infections. Although DNA viruses (e.g., HSV-1, adenoviruses) and RNA viruses (e.g., influenza A virus and SARS-CoV-2) trigger cGAS-STING signaling to mount essential antiviral defenses 24, 35, this evolutionary imperative pathway carries inherent risks. Excessive STING activation can drive cytokine storms and lead to ALI, exemplified in SARS-CoV-2 and influenza infections 36. Similarly, in pneumonia caused by* S. pneumoniae* infection, STING overactivation synergizes with MyD88-driven signaling to sustain the IFN-γ-mediated tissue damage 37. Notably, STING deficiency confers protection in polymicrobial sepsis models through decreasing bacteremia and pathogenic cytokine release 27. These findings collectively suggest that the hyperactivity of the cGAS-STING pathway often outweighs its defensive utility during severe viral and bacterial infection. Thus, it demands precise therapeutic strategies to preserve the baseline immunity while preventing excessive inflammation.

The clinical urgency of balancing this axis is especially magnified in acute respiratory distress syndromes (ARDS). The cGAS-STING hyperactivation within lung macrophages emerges as a unifying pathological mechanism across various ARDS triggers, including COVID-19, influenza pneumonia, and bacterial sepsis 11, 36, 38. Three convergent pathways contribute to the cGAS-STING dysregulation in macrophages: (i) mitochondrial distress caused by viral-induced release of mitochondrial DNA (mtDNA) into the cytosol activates STING, triggering massive production of type I IFNs that contribute to severe viral pneumonia. (ii) Bacteremia-induced cytolysis floods the macrophage cytoplasm with damage associated molecular patterns (DAMPs) to activate STING. (iii) A self-perpetuating cycle of cell necrosis and STING activation amplifies systemic inflammation and irreversible tissue damage. Therefore, pharmacological inhibition of the cGAS-STING pathway in macrophages represents a promising strategy to attenuate lethal inflammation and tissue injuries without severely compromising the host defense mechanism.

### Induction of autophagy-mediated STING degradation offers a novel way to modulate the cGAS-STING pathway

The cGAS-STING signaling can be down-regulated by autophagy-mediated degradation of STING. Specifically, STING can be directed by TBK1-mediated phosphorylation of p62 toward autophagic cargo receptor for autolysosomal degradation 18. In addition, chaperone-like proteins, such as UNC93B1 and UXT, have been identified to target STING for autophagic-lysosomal proteolysis 39, 40. Furthermore, the STING-containing endoplasmic reticulum (ER)-Golgi intermediate compartment (ERGIC) serves as a membrane scaffold for LC3 lipidation. This process initiates the formation of autophagosomes that subsequently fuse with lysosomes to degrade not only the STING protein but also the cGAS-STING signaling triggers, such as viral dsDNA or cytosolic dsDNA resulting from genomic instability and mitochondrial dysfunction 17. Thus, autophagy functions as a vital self-limiting mechanism that constrains STING-induced IFN responses to maintain immune homeostasis. Inspired by these facts, targeted degradation of STING through autophagy represents a dual-action therapeutic strategy: it dampens hyperactive cGAS-STING signaling and simultaneously facilitates the clearance of aberrant cytosolic dsDNA, thereby addressing both the driver and the consequence of the inflammatory response.

While exogenous molecular inducers of autophagy, such as rapamycin and metformin, have been proposed as potential therapeutic strategies to prevent excessive STING activation 41, 42, their clinical applications remain constrained. These small molecule agents often suffer from rapid systemic clearance, poor target selectivity, low bioavailability, and suboptimal biodistribution in diseased organs, limiting their therapeutic efficacy 43. To overcome these pharmacological hurdles, we developed CSE-P12, a unique, drug-free nanodevice designed to attenuate the cGAS-STING signaling pathway by potently triggering autophagy (**Figure 2**). Our transcriptomic analysis supports the robust autophagic response induced by CSE-P12: specifically, CSE-P12 significantly enhanced the “mitophagy animal” and “autophagy animal” pathways—an effect not observed with P12 or CSE treatment alone (**Figure 1D**). In addition, we observed specific up-regulation of the antigen processing and presentation pathways, further corroborating the induction of functional autophagy (**Figure 1D**). Importantly, CSE-P12 demonstrated significantly higher inhibitory activity on STING signaling in comparison with the classical autophagy inducer rapamycin (**Figure 2F and G**). Together, our findings demonstrate that the CSE-P12 nanodevice effectively regulate inflammatory signaling in macrophages by inducing autophagy to down-regulate STING. This mechanism likely facilitates the clearance of the bound, aberrant dsDNAs; it also highlights the distinct advantages of using engineered nanodevices for the precise modulation of immune signaling in complex inflammatory environments.

### The mechanisms of action of CSE-P12-induced autophagy for STING degradation

While we previously observed that CSE-P12 induces autophagy, the underlying molecular mechanism remained elusive 19. In this study, we defined the upstream signaling pathway responsible for this activity, identifying AMPK activation as the primary driver of CSE-P12-mediated autophagy (**Figure 7A-F**). AMPK is a crucial cellular energy sensor, and its activity is governed by the AMP-to-ATP or ADP-to-ATP ratio 44. Under conditions of energy depletion (less ATP), AMPK is activated to restore the energy homeostasis by upregulating ATP-producing catabolic processes while suppressing energy-consuming processes 45. We hypothesized that the massive internalization of CSE-P12 triggers acute metabolic stress. It is known that nanoparticle uptake is an inherently energy-intensive process, primarily occurring through endocytosis 46. We confirmed that CSE-P12 uptake is energy-dependent (**Figure 7G**), as TEM imaging revealed that nanoparticle internalization (indicated by red arrows) was significantly inhibited at 4°C (vs. 37°C) due to increased membrane rigidity and reduced metabolic activity. This rapid, large-scale uptake of CSE-P12 leads to a state of cellular energy exhaustion, which is validated by several lines of evidence: the significant increase in the ADP-to-ATP ratio was observed (**Figure 7H**); this metabolic shift resulted in the marked elevation of phosphorylated AMPK (**Figure 7I**); both autophagy induction and STING degradation were reversed by either pharmacological inhibition of AMPK or the addition of extracellular ATP (**Figure 7C-F**).

The energy-dependent AMPK activation has been reported in other types of nanoparticles 47. For example, the nano-zinc oxide was found to activate the AMPK-ULK1 signaling pathway through increasing the intracellular Ca^2+^ levels (CaMKK2 activation) and the AMP-to-ATP ratio 48. The mitochondria-targeted self-assembling nanoparticles were able to elevate the ADP-to-ATP ratio by directly inhibiting mitochondrial OXPHOS metabolism, leading to AMPK activation 49. Moreover, the RGD peptide-coated nanoparticles encapsulating metformin and glucose oxidase exhibited the ability to suppress glycolysis to activate AMPK 50. Ours and these lines of evidence suggest that the metabolic demand of nanoparticle internalization can be leveraged as a “molecular switch” for AMPK activation.

Overall, CSE-P12 functions as a nanoscale STING inhibitor by triggering autophagic degradation of STING in macrophages. The energy-dependent uptake of CSE-P12 shifts the ADP/ATP ratio and hance activates the AMPK-autophagy axis. These insights provide a novel framework for designing drug-free nanotherapeutics that modulate immune signaling through intrinsic metabolic pathways.

### CSE-P12 as a new generation of nanomedicine for infectious inflammatory lung diseases

Nanoscale STING inhibitors have become a new class of anti-inflammatory therapies, offering superior cellular targeting and tissue distribution to systemic small molecules. Recent advances have yielded several promising candidates: Polyethyleneimine-coated mesoporous polydopamine nanoparticles loaded with STING antagonist C-176 (PEI-PDA@C-176 NPs) effectively inhibited the cGAS-STING pathway in macrophages for treating rheumatoid arthritis 51. Liposomes carrying both the NF-κB suppressor MLN4924 and STING inhibitor H-151 (Lip@MH) targeted macrophages and synergistically inhibited both pathways to down-regulate inflammatory cytokine production 52. Nano-micelles loaded with STING inhibitor RU.521 were developed for colitis treatment 53. Dextran core nanoparticles loaded with GSK-J1 inhibitors targeted macrophages and ameliorated chronic inflammation mediated by NF-κB and STING pathways in diabetic wounds 54. Although these platforms demonstrate the efficacy of macrophage-specific STING inhibition, they often rely on complex multi-component designs with drug encapsulation. Such complexity poses significant hurdles for large-scale manufacturing and clinical translation.

Departing from traditional nanotherapeutics that serve as mere vehicles for drug cargo, we developed **CSE-P12**—a fundamentally different, drug-free bioactive nanodevice. Comprising gold nanoparticles functionalized with bioactive peptides and trace CSE, CSE-P12 offers several distinct advantages for the management of infectious inflammatory lung diseases. First, CSE-P12 can target pulmonary macrophages to regulate the cGAS-STING pathway, a central driver in the pathogenesis of viral and bacterial pneumonia (**Figure S1, Figures 3-6, Figure S11**). Second, built from three components (GNPs, hexapeptides, and 1% CSE), this nanodevice is amenable to standardized, large-scale production. Third, unlike single-target inhibitors, CSE-P12 exerts a multimodal mechanism of action, simultaneously attenuating both cGAS-STING and TLR signaling pathways to achieve comprehensive immune resolution. Fourth, the macrophage-specific STING modulation allows for location-adapted dosing regimens to achieve spatiotemporal precision intervention that systemic inhibitors cannot match. The physiological stability and favorable biosafety profile of CSE-P12 make it a robust candidate for treating infection-associated inflammatory complications.

## Conclusions

In this study, we demonstrated that the CSE-functionalized nanodevice CSE-P12 served as a potent modulator of the cGAS-STING pathway to effectively suppress pathological IFN responses and downstream hyper-inflammation in macrophages. By leveraging autophagy-mediated STING degradation, CSE-P12 ameliorated lung inflammation and injuries in HAdV4 virus infection and CLP-induced bacterial sepsis mouse models. Our mechanistic investigations revealed that the anti-inflammatory action of CSE-P12 was driven by the energy-dependent internalization of the nanodevice, leading to a localized elevation of the ADP-to-ATP ratio within macrophages. This metabolic shift triggered the AMPK activation that induced autophagy to clear STING proteins and dampen the associated inflammatory responses. Overall, this work introduces a novel, cargo-free nanotherapeutic strategy for the precision management of aberrant cGAS-STING signaling. By targeting the autophagic machinery of macrophages, CSE-P12 offers a precision intervention for treating viral pneumonia, sepsis-associated lung injury, and a broader spectrum of inflammatory respiratory diseases.

## Supplementary Material

Supplementary figures and tables, methods, including single-cell transcriptomics analysis, the evidence of the lack of binding between CSE-P12 and cGAMP, the effects of CSE-P12 on TNF-α and autophagic flux in macrophages, the protective effects of CSE-P12 on DNA virus HAdV4- and influenza virus PR8-induced lung injury, the gating strategy of flow cytometry analysis, the alveolar and interstitial neutrophil scores in the HAdV4-infected mouse lungs, the therapeutic effects of CSE-P12 in CLP-induced sepsis mouse model in wild-type and *Sting*^-/-^ mice, and the effects of CSE-P12 on the phosphorylation of AKT and mTOR in macrophages.

## Data Availability

All data generated and analyzed during this research are included in this article and available upon request.
