# Supplementary material for: Attenuation of cGAS-STING signaling-mediated lung inflammation during infection through autophagy induction by bioactive nanodevices
Source: Theranostics. 2026 Mar 4;16(10):5204–25. doi: 10.7150/thno.127113 (PMC13080449; doi:10.7150/thno.127113)
Supplement: Supplementary file 1 — Supplementary figures and tables, methods, including single-cell transcriptomics analysis, the evidence of the lack of binding between CSE-P12 and cGAMP, the effects of CSE-P12 on TNF-α and autophagic flux in macrophages, the protective effects of CSE-P12 on DNA virus HAdV4- and influenza virus PR8-induced lung injury, the gating strategy of flow cytometry analysis, the alveolar and interstitial neutrophil scores in the HAdV4-infected mouse lungs, the therapeutic effects of CSE-P12 in CLP-induced sepsis mouse model in wild-type and Sting-/- mice, and the effects of CSE-P12 on the phosphorylation of AKT and mTOR in macrophages. [file thnov16p5204s1.pdf]

# Supporting Information

## Attenuation of cGAS-STING signaling-mediated lung inflammation during infection through autophagy induction by bioactive nanodevices

Mimi Pang[1,#], Xiang Wang[2,#], Zichen Song[3,#], Rujing Lin[1], Sixia Liu[1], Man Xing[4], Wenfei Xu[1], Jiameng Gong[1], Ying Qi[5], Mei Du[7], Yonghao Yu[3], Bing Chen[5,✉], Shan-Yu Fung[6,7,✉], Dongming Zhou[2,4,✉], Hong Yang[1,7,✉]

1 Department of Pharmacology and Tianjin Key Laboratory of Inflammation Biology, The Province and Ministry Co-Sponsored Collaborative Innovation Center for Medical Epigenetics, School of Basic Medical Sciences, Intensive Care Unit of the Second Hospital, Tianjin Medical University, Tianjin 300070, China

2 Shanghai Public Health Clinical Center, Fudan University, Shanghai, 201508, China

3 Department of Anesthesia, Tianjin Institute of Anesthesiology, Tianjin Medical University General Hospital, Tianjin 300052, China

4 Department of Pathogen Biology, School of Basic Medical Sciences, Tianjin Medical University, Tianjin 300070, China

5 Institute of Infectious Diseases, Intensive Care Unit of the Second Hospital, Tianjin Medical University, Tianjin 300211, China

6 State Key Laboratory of Experimental Hematology, Department of Immunology and Key Laboratory of Immune Microenvironment and Disease (Ministry of Education), School of Basic Medical Sciences, Tianjin Medical University, Tianjin 300070, China

7 International Joint Laboratory of Ocular Diseases (Ministry of Education), Tianjin Key Laboratory of Ocular Trauma, Tianjin Medical University, Tianjin 300052, China

# These authors contributed equally to this work.

✉ Corresponding authors: Hong Yang (hongyang@tmu.edu.cn), Dongming Zhou (zhoudongming@tmu.edu.cn), Shan-Yu Fung (shanefung@tmu.edu.cn), Bing Chen (tisheng2008@163.com).

**List of contents for supporting information**

**S1. Supplementary methods**

**S1.1 Single-cell RNA sequencing data analysis**

**S1.2 The effects of CSE-P12 and cGAMP interaction on cGAMP-mediated activation of NF-κB/AP-1 and IRF**

**S1.3 Immunoblotting analysis**

**S1.4 Autophagic flux by confocal imaging**

**S2. Supplementary tables**

**Table S1:** List of primer sequences for measuring the mRNA levels of IFN-stimulated genes and pro-inflammatory cytokine genes by RT-qPCR.

**Table S2:** List of primer sequences for measuring the HAdV4 and PR8 viral loads by RT-qPCR.

**S3. Supplementary figures**

**Figure S1**: Single-cell transcriptomics reveals the increased population and inflammatory responses of monocytes and macrophages in the mouse lung post-influenza infection.

**Figure S2**: The effects of CSE-P12 on the transcriptomic profile in THP-1 cell-derived macrophages stimulated with cGAMP.

**Figure S3**: The inhibitory activity of CSE-P12 on cGAS-STING signaling pathway was not through cGAMP binding.

**Figure S4**: The inhibitory activity of CSE-P12 on the production of type I IFNs and pro-inflammatory cytokines downstream of the cGAS-STING signaling pathway.

**Figure S5**: Densitometry analysis of the protein bands in the immunoblots presented in Figure 1K.

**Figure S6**: The effects of the CSE-P12 or rapamycin treatment on the autophagic flux in THP-1 LC3 reporter cell-derived macrophages.

**Figure S7**: The effects of CSE-P12 on the cGAMP-induced mRNA levels of IFNB1 by RT-qPCR in THP-1-derived macrophages.

**Figure S8**: Densitometry analysis on the protein bands in the immunoblots presented in Figure 2I.

**Figure S9**: The effect of CSE-P12 on the body weight and viral load in mice induced by DNA virus HAdV4.

**Figure S10**: The gating strategy of flow cytometry analysis to identify different immune cells

in the lung.

**Figure S11**: The protective effects of CSE-P12 on influenza virus PR8 infection-induced lung inflammation and injury in mice.

**Figure S12**: The effect of CSE-P12 on the body weight and bacterial loads in the CLP-induced sepsis mouse model.

**Figure S13**: Densitometry analysis on the protein bands in the immunoblots presented in Figure 6C.

**Figure S14**: Therapeutic effects of CSE-P12 in CLP-induced sepsis mouse model.

**Figure S15**: The effects of CSE-P12 on the pathophysiological characteristics of CLP-induced lung injury in wild-type and $STING^{-/-}$ mice.

**Figure S16**: The effects of CSE-P12 on the phosphorylation of mTOR and AKT in THP-1 cell-derived macrophages.

**Figure S17**: Densitometry analysis on the protein bands in the immunoblots presented in Figure 7C and E.

## S1. Supplementary methods

### S1.1. Single-cell RNA sequencing data analysis

Single-cell RNA sequencing data were obtained from the GEO data resource (GSE202325 and GSE124872). Seurat (Version 5.3.0) in R (Version 4.5.0) was used for dimensional reduction, clustering and data analysis.

### S1.2. The effects of CSE-P12 and cGAMP interaction on cGAMP-mediated activation of NF-κB/AP-1 and IRF

To rule out the possibility that the inhibitory activity of nanoparticles on cGAS-STING signaling may be affected by CSE-P12 and cGAMP interaction, cGAMP was co-incubated with P12 or CSE-P12 for 2 h, and the supernatants were collected after centrifugation. THP-1 reporter cell-derived macrophages were stimulated with cGAMP and treated with/without the supernatants or P12 and CSE-P12 (for comparison) for 24 h. The culture medium was then collected and incubated with QUANTI-Blue solution until the solution color turned into dark blue. The absorption at 655 nm was measured by a microplate reader (TECAN, Mannedorf, Zurich, Switzerland).

### S1.3 Immunoblotting analysis

To study the effect of autophagy inhibitor 3-MA on autophagy induced by CSE-P12, the THP-1 cell-derived macrophages were pretreated with 3-MA (10 μM) for 1 h, followed by

cGAMP (5 μg/mL) stimulation with/without CSE-P12 treatment for 4 h.

To explore the effect of CSE-P12 on mTOR signaling pathway, the THP-1 cell-derived macrophages were stimulated with cGAMP with or without P12 (100 nM), 1% CSE, and CSE-P12 (100 nM) for different periods up to 1 h. The cell lysate preparation and immunoblotting experiments were performed using the same procedures described in the main text.

### S1.4 Autophagic flux by confocal imaging

The THP-1 LC3 reporter cell-derived macrophages expressing the RFP-GFP-LC3 fusion protein were treated with cGAMP (5 μg/mL) with/without CSE-P12 or rapamycin (10 μM) treatment for 12 h, and then imaged by a confocal microscopy (Olympus, Japan). The numbers of RFP and GFP punctate dots were counted in each cell (> 45 cells for each sample), and averaged from three independent experiments.

## S2. Supplementary tables

**Table S1:** List of primer sequences for measuring the mRNA levels of IFN-stimulated genes and pro-inflammatory cytokine genes by RT-qPCR.

| Name | Forward | Reverse |
|---|---|---|
| H-MX1 | CTCCGACACGAGTTCCACAA | ATCTGTGAAAGCAAGCCGGA |
| H-CXCL10 | TGGCATTCAAGGAGTACCTC | TTGTAGCAATGATCTCAACACG |
| H-ISG15 | CGCAGATCACCCAGAAGATCG | TTCGTCGCATTTGTCCACCA |
| M-Ifnb1 | ATGAGTGGTGGTTGCAGGC | TGACCTTTCAAATGCAGTAGATTCA |
| M-ISG15 | AGAAGCAGATTGCCCAGAAG | TGCGTCAGAAAGACCTCATAGA |
| M-IL-6 | GCCTTCTTGGGACTGATGCT | TGTGACTCCAGCTTATCTCTTGG |
| M-IL-12b | CCTGTGACACGCCTGAAGAAGATG | CTTGTGGAGCAGCAGATGTGAGTG |

**Table S2:** List of primer sequences for measuring the HAdV4 and PR8 viral loads by RT-qPCR.

| Name | Forward | Reverse |
|---|---|---|
| Hexon gene | ACCAGCTCTTGCTTGACTCT | GGCAATTCATCCTCCACACC |
| M gene | AAGACCAATCCTGTCACCTCTGA | CAAAGCGTCT ACGCTGCAGTCC |

## S3. Supplemental figures

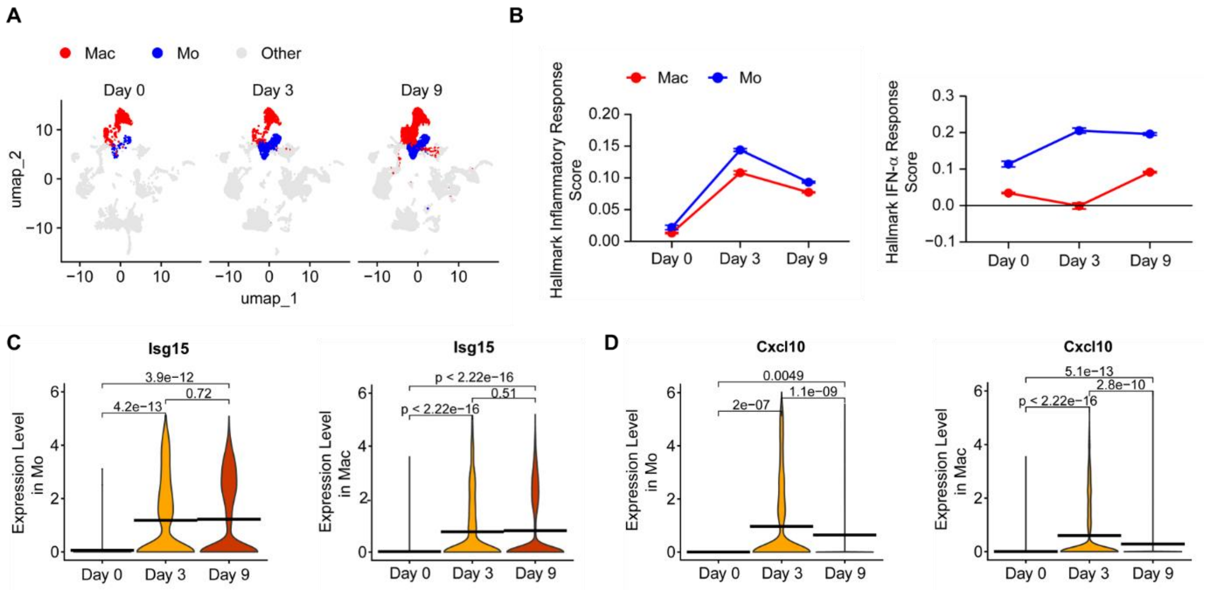


**Figure S1. Single-cell transcriptomics reveals the increased population and inflammatory responses of monocytes and macrophages in the mouse lung post-influenza infection.** (A) UMAP plot for monocytes (Mo), macrophages (Mac) and other cells in the lungs from mice on Day 3 and Day 9 post-infection. (B) Line charts showing module scores of the Hallmark Inflammatory Response gene set (GSEA systematic name MM3890; left) and Hallmark Interferon Alpha Response gene set (MM3877; right) in monocytes and macrophages from the mouse lungs on Day 3 and Day 9 post-infection. (C-D) Violin plots showing expression of *Isg15* (C) and *Cxcl10* (D) in monocytes (left) and macrophages (right) from the mouse lungs on Day 3 and Day 9 post-infection; the horizontal lines denote mean expression values.

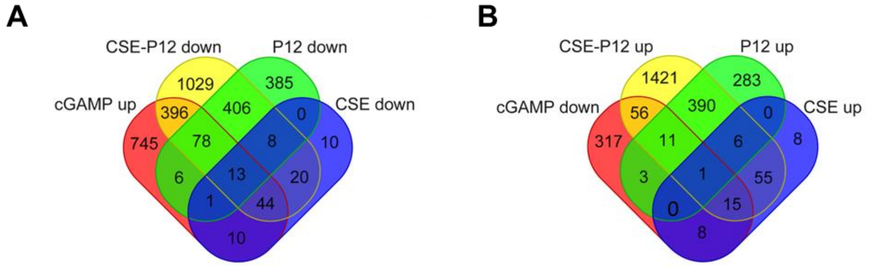


**Figure S2. The effects of CSE-P12 on the transcriptomic profile in THP-1 cell-derived macrophages stimulated with cGAMP.** (A, B) The Venn diagram showing the number of cGAMP up-regulated (A) and down-regulated (B) genes that were attenuated by P12, CSE and CSE-P12 treatments in THP-1 cell-derived macrophages; $p < 0.05$ and $|\log2\ (fc)| > 1$ as the cut-off criteria; cells were stimulated with cGAMP with or without different treatments for 6 h.

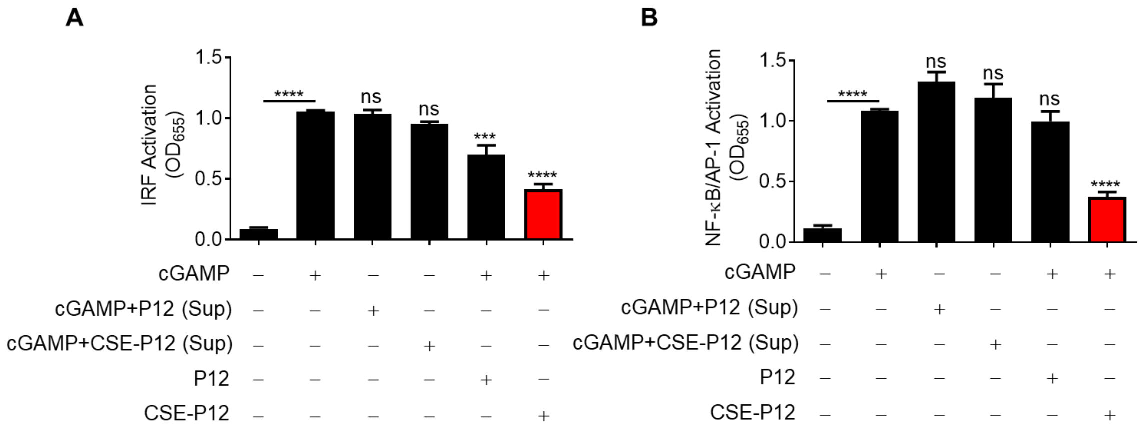


**Figure S3. The inhibitory activity of CSE-P12 on cGAS-STING signaling pathway was not through cGAMP binding.** (A, B) The effects of the supernatants of the cGAMP+P12 or cGAMP+CSE-P12 mixtures (incubation time = 2 h) after centrifugation on the activation of IRF (A) and NF-κB/AP-1 (B) in comparison with the cGAMP stimulation with/without P12 and CSE-P12 treatments in the THP-1 reporter cell-derived macrophages; N = 3. cGAMP = 5 μg/ mL, P12 = 100 nM, CSE-P12 = 100 nM. The data is presented as mean ± SEM; ns: not significant, ***p < 0.001, ****p < 0.0001 vs. cGAMP stimulation unless otherwise indicated.

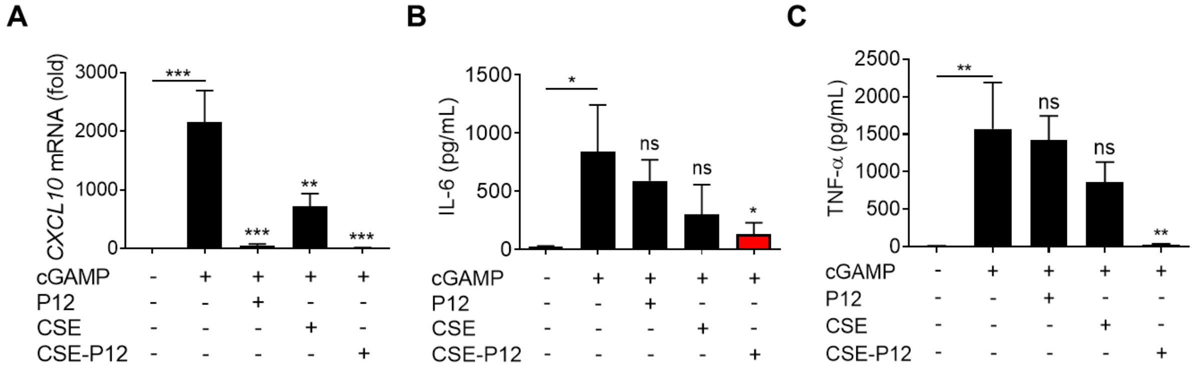


**Figure S4. The inhibitory activity of CSE-P12 on the production of type I IFNs and pro-inflammatory cytokines downstream of the cGAS-STING signaling pathway.** (A-C) The effects of CSE-P12 on the mRNA levels of *Cxcl10* (A) by RT-qPCR as well as the protein levels of IL-6 (B) and TNF-α (C) by ELISA upon cGAMP stimulation for 24 h in THP-1 cell-derived macrophages; N = 4. cGAMP = 5 μg/ mL, P12 and CSE-P12 = 100 nM, CSE = 1%. The data is presented as mean ± SEM, ns: not significant, *p < 0.05, **p < 0.01, ***p < 0.001 vs. cGAMP stimulation group unless otherwise indicated.

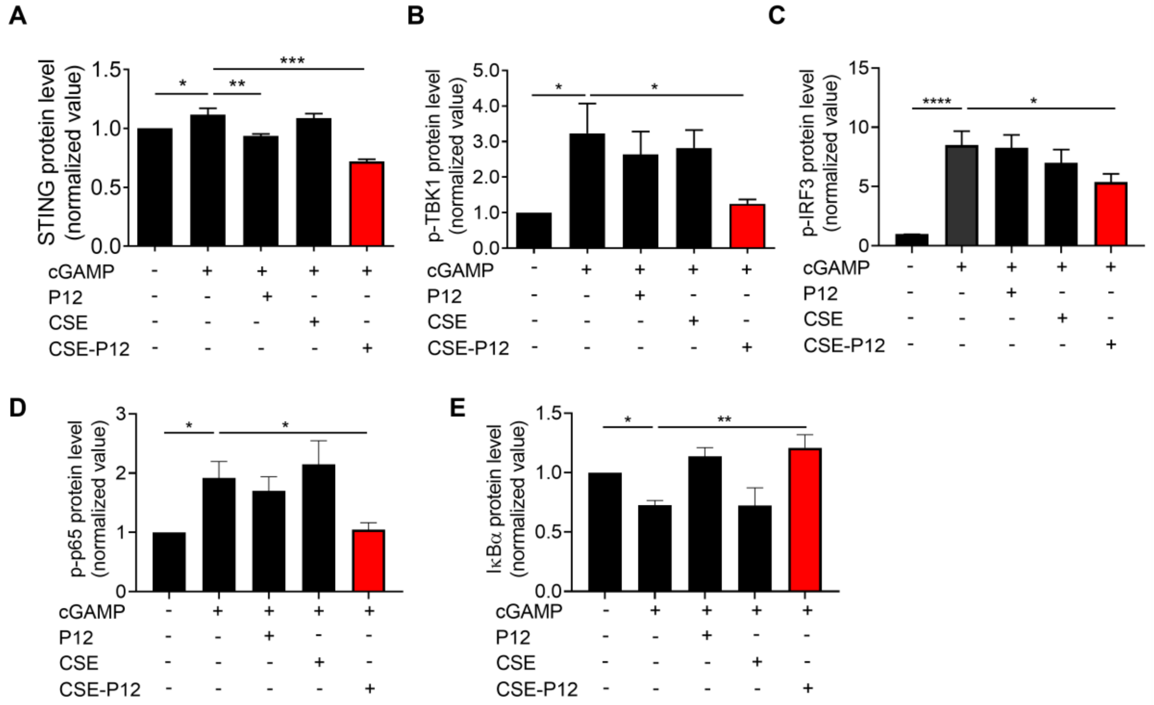


**Figure S5. Densitometry analysis of the protein bands in the immunoblots presented in Figure 1K.** (A-E) Densitometry analysis showing the levels of STING (A), phosphorylated TBK1 (p-TBK1) (B), phosphorylated IRF3 (p-IRF3) (C), phosphorylated p65 (p-p65) (D), and IκBα (E) from the immunoblots in Figure 1K; all protein levels were normalized to the internal control β-actin and the unstim group; $N \geq 3$. The data is presented as mean ± SEM; ns: not significant, $^{*}p < 0.05$, $^{**}p < 0.01$, $^{***}p < 0.001$, $^{****}p < 0.0001$.

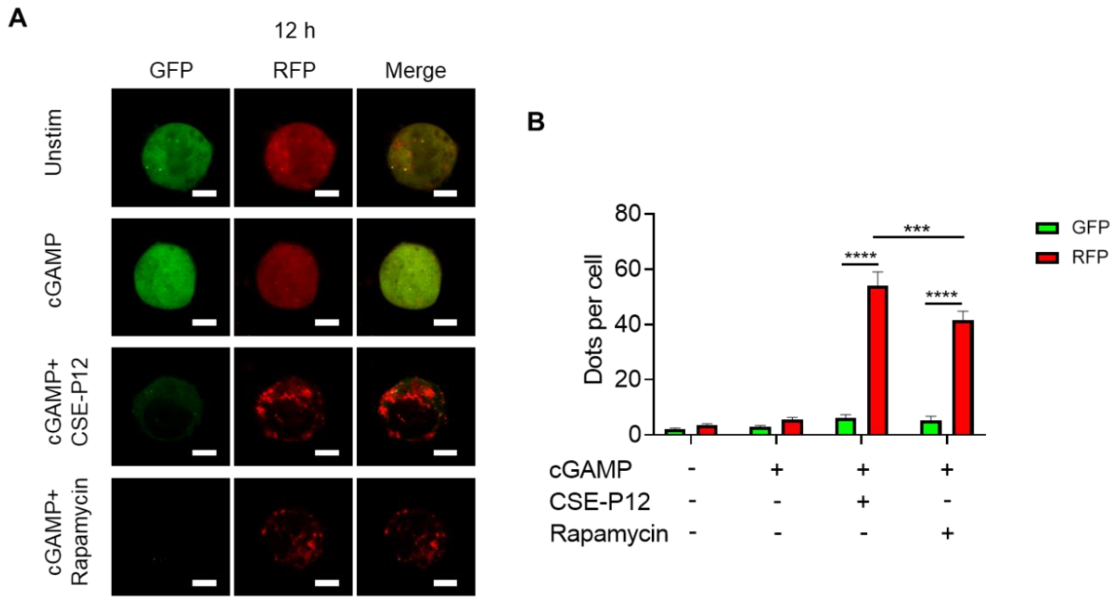


**Figure S6. The effects of the CSE-P12 or rapamycin treatment on the autophagic flux in THP-1 LC3 reporter cell-derived macrophages.** (A) Representative confocal images of the RFP-GFP-LC3 fusion protein in the THP-1 LC3 reporter cell-derived macrophages treated with CSE-P12 and rapamycin under cGAMP stimulation for 12 h; scale bar = 5 μm. (B) Average numbers of RFP (red) and GFP (green) dots per cell in each group quantified from (A); more than 45 cells were examined for each group. CSE-P12 = 100 nM, rapamycin =10 μM, cGAMP = 5 μg/mL; N = 3; ***p < 0.001, ****p < 0.0001.

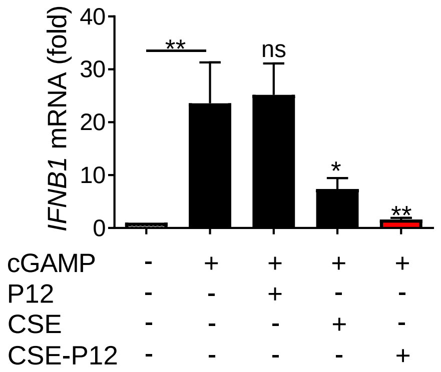


**Figure S7. The effects of CSE-P12 on the cGAMP-induced mRNA levels of *IFNB1* by RT-qPCR in THP-1-derived macrophages.** Cells were stimulated with cGAMP for 6 h with P12, CSE or CSE-P12 co-treatment. N = 4; cGAMP = 5 μg/ mL, P12 and CSE-P12 = 100 nM, CSE = 1%. The data is presented as mean ± SEM, ns: not significant, *p < 0.05, **p < 0.01 vs. The cGAMP stimulation group unless otherwise indicated.

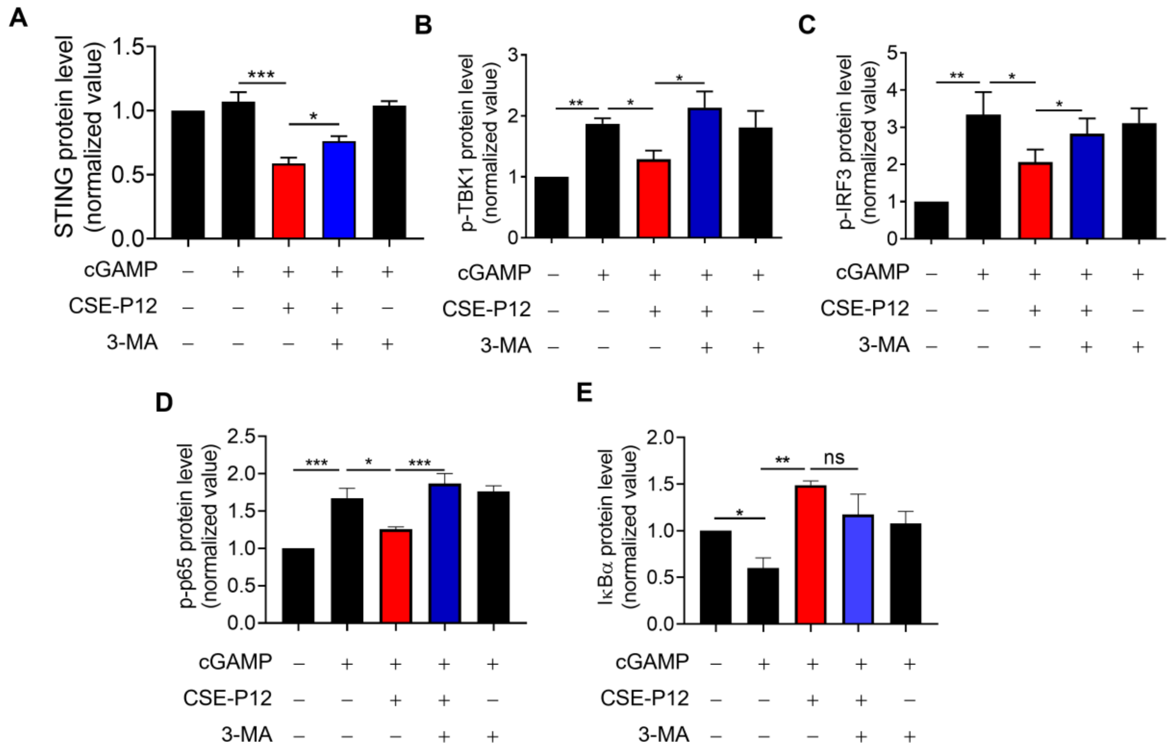


**Figure S8. Densitometry analysis on the protein bands in the immunoblots presented in Figure 2I.** (A-E) Densitometry analysis on the levels of STING (A), phosphorylated TBK1 (p-TBK1) (B), phosphorylated IRF3 (p-IRF3) (C), phosphorylated p65 (p-p65) (D), and IκBα (E) from the immunoblots in Figure 2I; all protein levels were normalized to the internal control β-actin or GAPDH and the unstim group; $N \geq 3$. The data is presented as mean ± SEM; ns: not significant, *$p < 0.05$, **$p < 0.01$, ***$p < 0.001$.

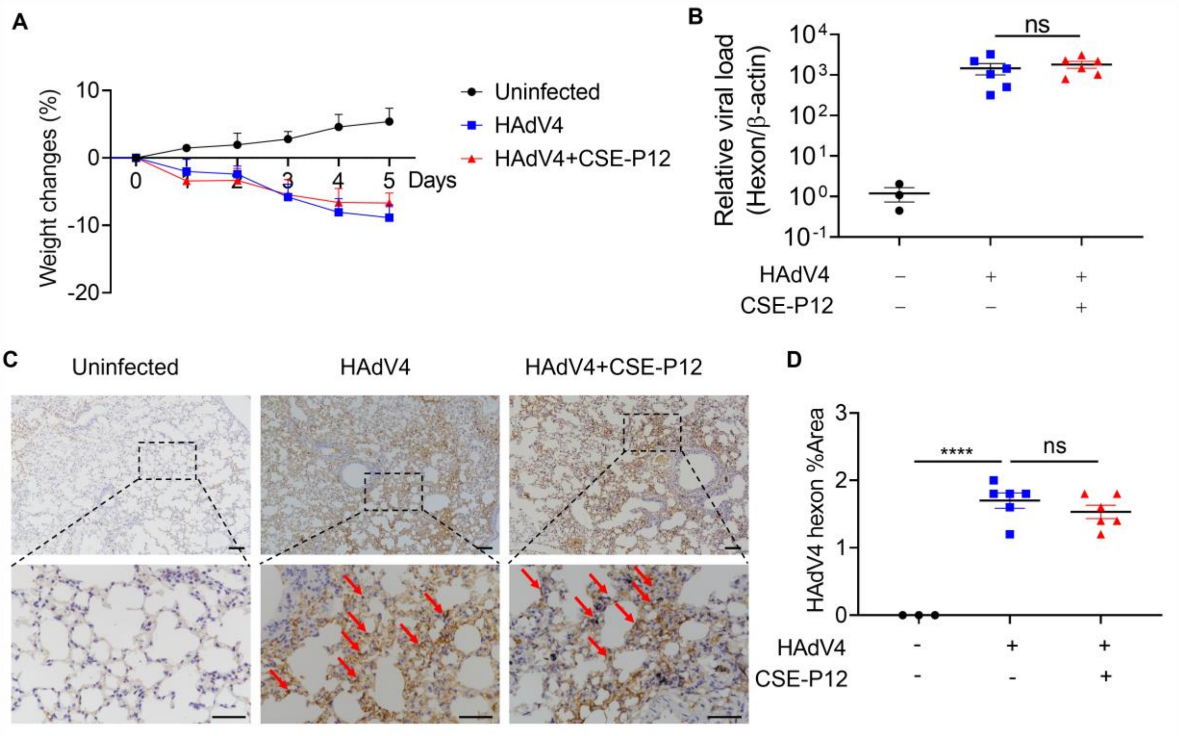


**Figure S9. The effect of CSE-P12 on the body weight and viral load in mice induced by DNA virus HAdV4.** (A) Body weight changes in mice after HAdV4 infection. (B)The viral titer in the lung tissues of mice from uninfected (PBS), HAdV4 infected, and HAdV4+CSE-P12 groups by RT-qPCR analysis on the Hexon gene expression of HAdV4; β-actin as the intrinsic reference gene. (C) Representative IHC staining images of HAdV4 protein Hexon; Scale bar = 100 μm. (D) Quantitative analysis of Hexon protein from the IHC images of the groups in (C); PBS group: N = 3, HAdV4 group: N = 6, HAdV4+CSE-P12 group: N = 6; The data is presented as mean ± SEM; ns: not significant, $*p < 0.05$, $**p < 0.01$, $***p < 0.001$, $****p < 0.0001$.

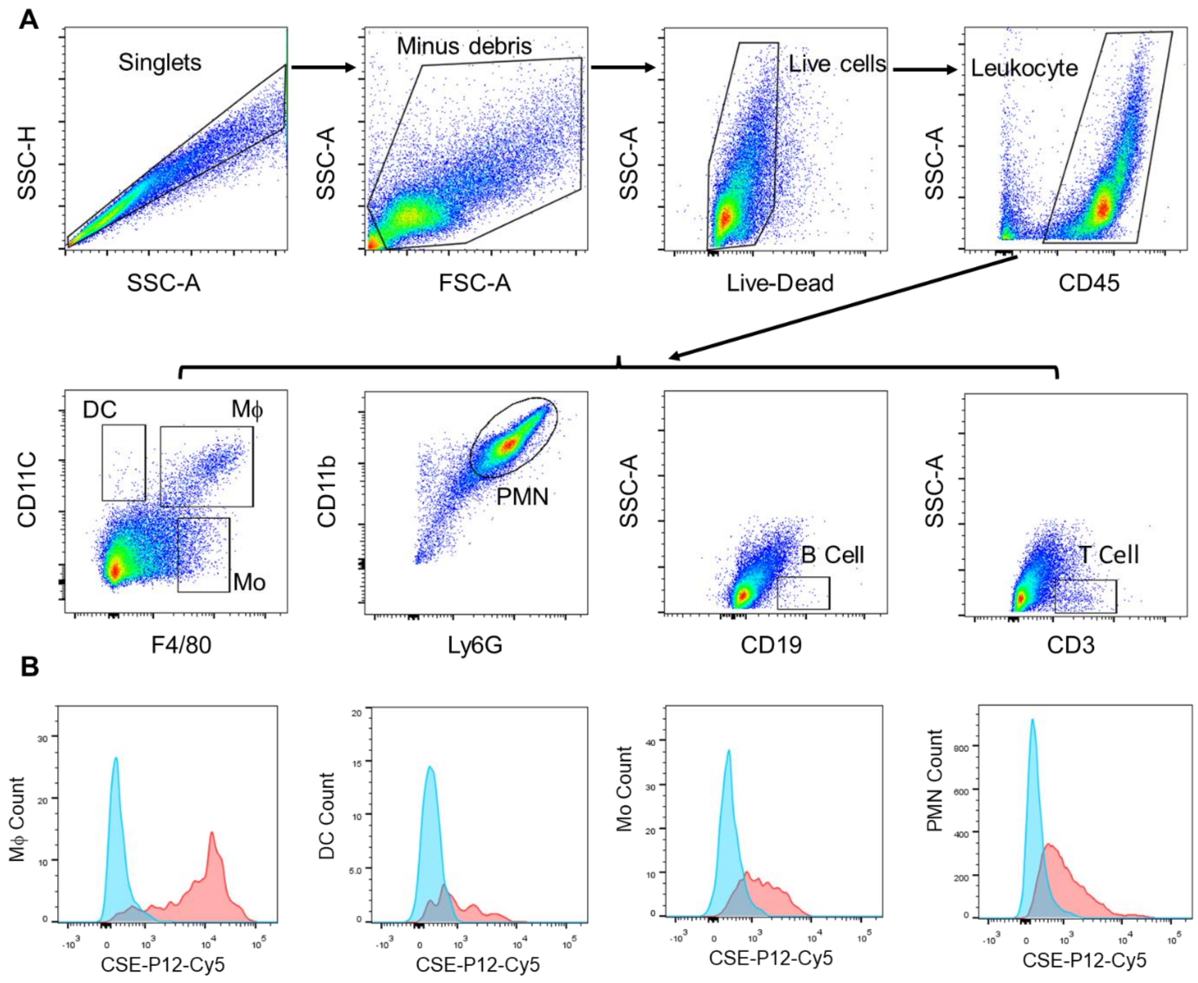


**Figure S10. The gating strategy of flow cytometry analysis to identify different immune cells in the lung.** (A) Gating strategy for the flow cytometry analysis to identify monocytes (Mo), dendritic cells (DCs), macrophages (Mϕ), neutrophils (PMN), T cells and B cells in the BALF. (B) Histogram plots showing the uptake of CSE-P12-Cy5 nanoparticles in various phagocytic immune cells: macrophages, DCs, monocytes and neutrophils; the CSE-P12-Cy5 treated group shown in red and the HAdV4 infected control group (without treatment) shown in blue.

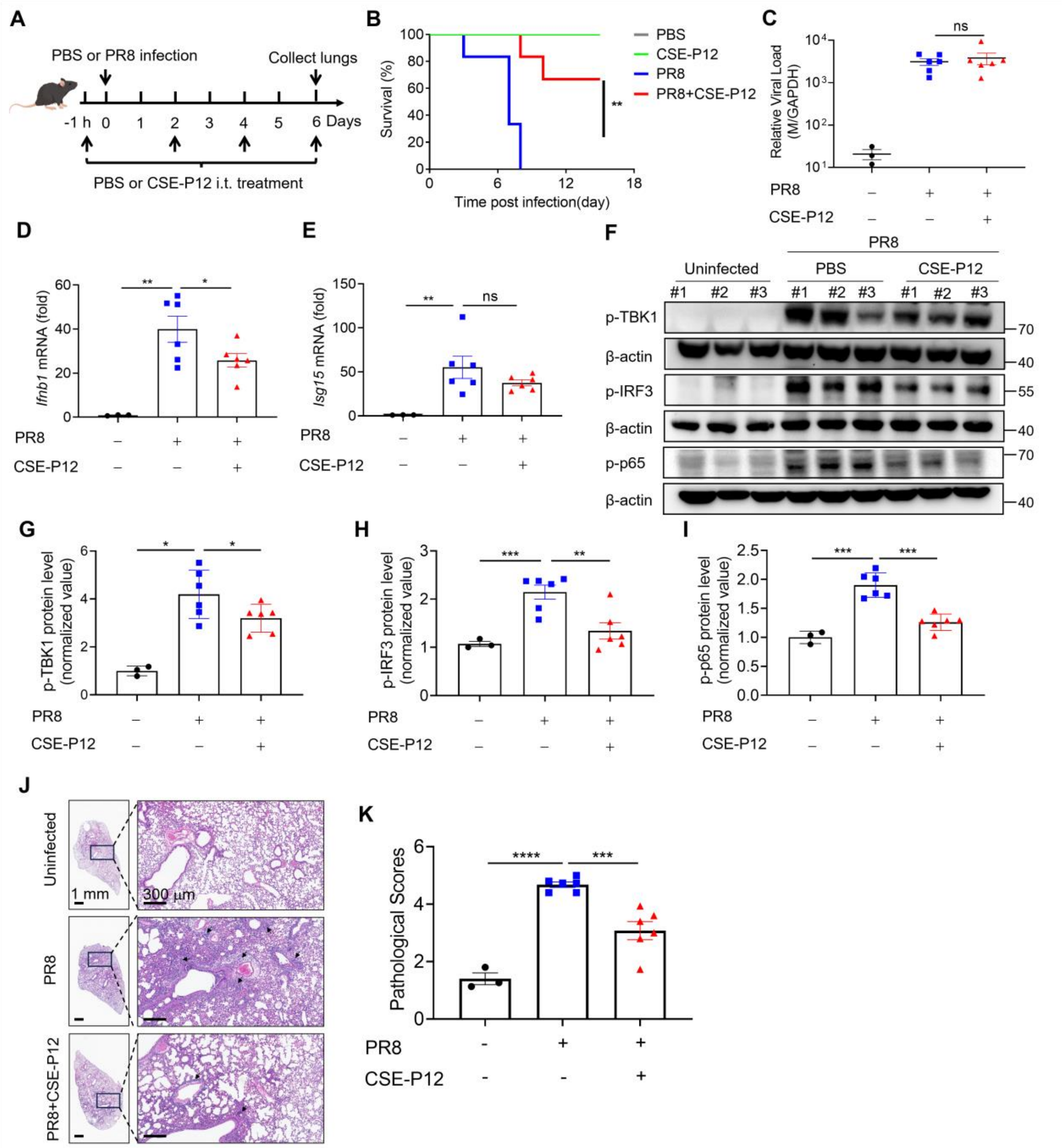


**Figure S11. The protective effects of CSE-P12 on influenza virus PR8 infection-induced lung inflammation and injury in mice.** (A) A scheme showing the PR8-induced pneumonia mouse model; mice were infected with PR8 (intranasal instillation, 5500 PFU per mouse) and given 4 doses of CSE-P12 treatment (500 nM, 50 μL) through intratracheal injection (i.t.) at -1 h and on Day 2, 4 and 6; mice were sacrificed on Day 6 (1 h after the last CSE-P12 treatment) for further analysis. (B) The survival rate of mice over 14 days following the same treatment as in (A); N = 6 mice per group. (C) The viral titer in the lung tissues of mice from the uninfected, PR8 infected, and PR8+CSE-P12 groups by RT-qPCR analysis on the M gene expression of PR8; GAPDH as the intrinsic reference gene. (D, E) The mRNA levels of the *Ifnb1* (D) and *Isg15* (E) genes in the lung of mice from the above 3 groups by RT–qPCR; GAPDH as the

intrinsic reference gene. (F) Immunoblots showing the levels of p-TBK1, p-IRF3, and p-p65 downstream the cGAS-STING pathway in the lung tissues of mice from the above 3 groups; β-actin as the internal control. (G-I) Densitometry analysis on the levels of p-TBK1 (G), p-IRF3 (H), and p-p65 (I) in (F); N = 3 for uninfected group and N = 6 for PR8 infected and PR8+CSE-P12 groups. (J) The representative H&E images of the lung sections from the above 3 groups; scale bar = 1 mm (left) and 300 μm (right). (K) Histological score of lung tissue quantified from the H&E images in (j); N = 3 for uninfected group and N = 6 for PR8 infected and PR8+CSE-P12 groups. The data is presented as mean ± SEM; ns: not significant, *p < 0.05, **p < 0.01, ***p < 0.001.

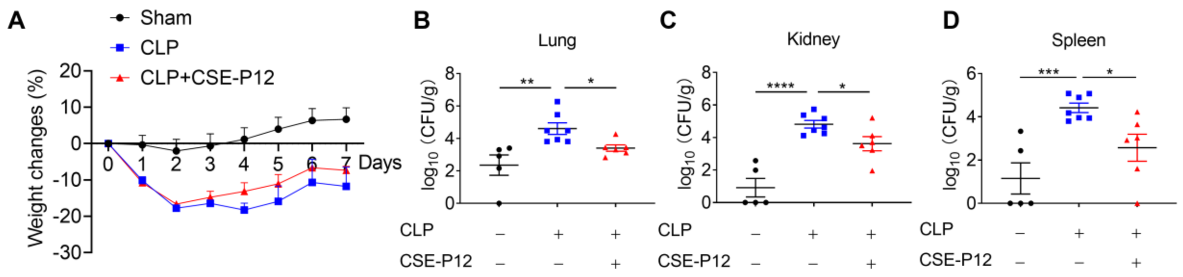


**Figure S12. The effects of CSE-P12 on the body weight and bacterial loads in the CLP-induced sepsis mouse model.** (A) Body weight changes in CLP-induced sepsis mouse model; N = 6 for the sham group, N = 12 for the CLP and CLP+CSE-P12 groups. (B-D) Effects of CSE-P12 on the bacterial loads in the lung (B), kidneys (C), and spleen (D) of CLP-induced sepsis mice; CSE-P12 (500 nM, 100 μL) or PBS treatment through i.p injection. The data is presented as mean ± SEM; ns: not significant, *p < 0.05, **p < 0.01, ***p < 0.001.

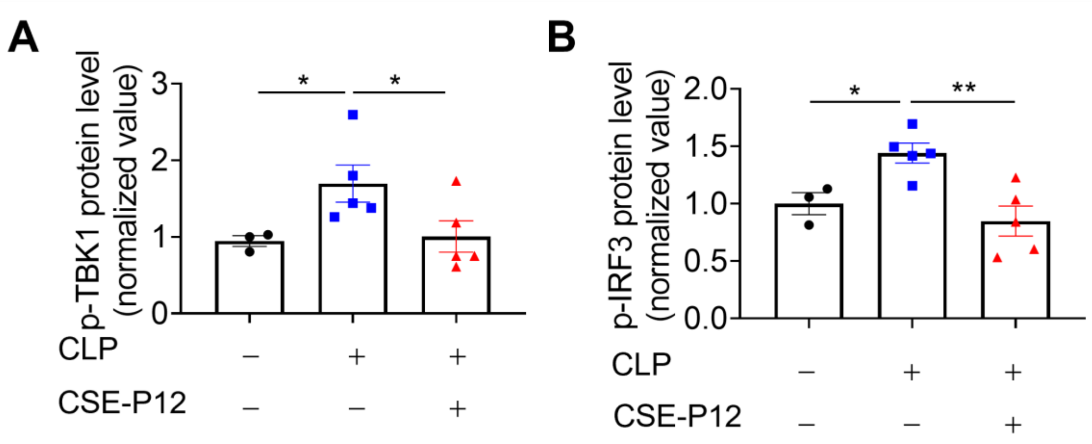


**Figure S13. Densitometry analysis on the protein bands in the immunoblots presented in Figure 6C.** (A, B) Densitometry analysis of p-TBK1 (A) and p-IRF3 (B) in the immunoblots in Figure 6C; N ≥ 3. The data is presented as mean ± SEM; ns: not significant, *p < 0.05, **p < 0.01, ***p < 0.001.

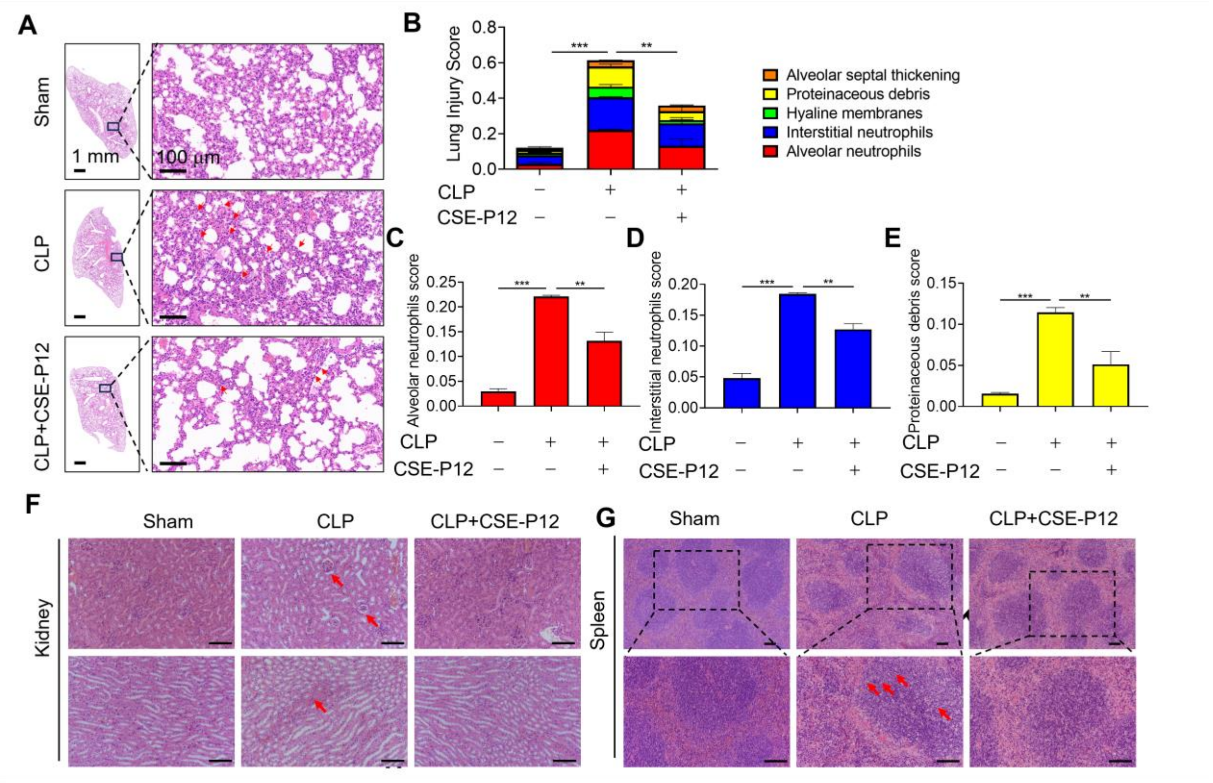


**Figure S14.Therapeutic effects of CSE-P12 in CLP-induced sepsis mouse model.** (A) The representative H&E images of lung sections of CLP-induced sepsis mice with or without the CSE-P12 treatement; scale bar = 1 mm (left) and 100 μm (right); red arrows indicated the infiltration of inflammatory cells. (B) The lung injury scores analyzed based on the 5 pathophysiological features in the H&E images in (A). (C-E) The lung injury scores of the specific pathophysiological features: alveolar neutrophils (C), interstitial neutrophils (D), and proteinaceous debris (E); N ≥ 3. (F, G) The representative H&E images of the kidney (F) and spleen (G) under CLP induction for 3 days with or without the CSE-P12 treatment; the red arrows indicated the site of the pathological lesions; scale bar = 100 μm. The data is presented as mean ± SEM; *$p < 0.05$, **$p < 0.01$, ***$p < 0.001$.

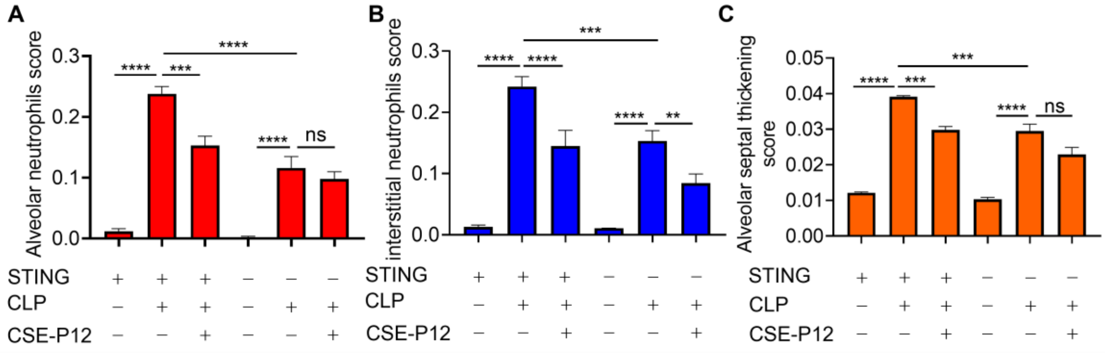


**Figure S15. The effects of CSE-P12 on the pathophysiological characteristics of CLP-induced lung injury in wild-type and STING$^{-/-}$ mice.** (A-C) The lung injury score of the pathophysiological characteristics of alveolar neutrophils (A), interstitial neutrophils (B), and alveolar septal thickening (C) in Figure 6I. N ≥ 3; The data is presented as mean ± SEM; ns: not significant, **p < 0.01, ***p < 0.001, ****p < 0.0001.

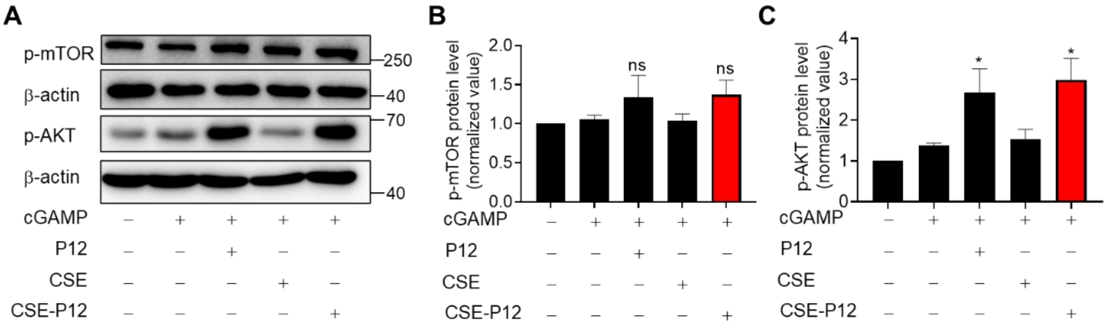


**Figure S16. The effects of CSE-P12 on the phosphorylation of mTOR and AKT in THP-1 cell-derived macrophages.** (A) Immunoblots showing the effects of CSE-P12 on the phosphorylation of mTOR (p-mTOR) and AKT (p-AKT) in THP-1 cell-derived macrophages under cGAMP stimulation for 1 h; β-actin as the internal control. (B, C) Densitometry analysis on the levels of p-mTOR (B) and p-AKT (C) in the immunoblots in (A). cGAMP = 5 μg/mL, P12 = 100 nM, CSE = 1%, CSE-P12 = 100 nM. N = 3; The data is presented as mean ± SEM; ns: not significant, *p < 0.05 vs. the cGAMP group.

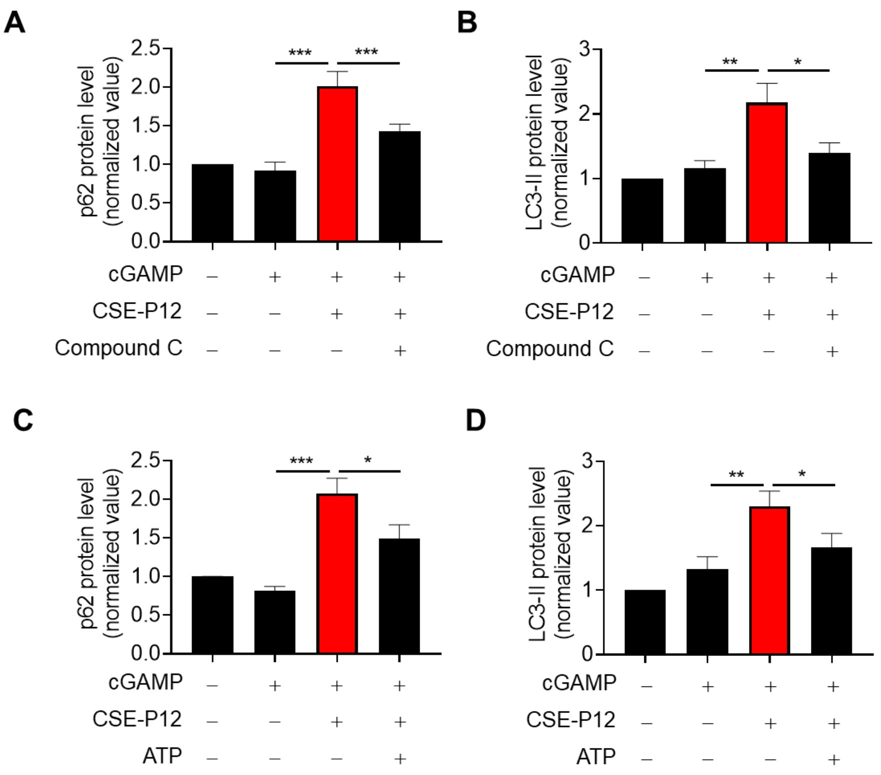


**Figure S17. Densitometry analysis on the protein bands in the immunoblots presented in Figure 7C and E.** (A-B) Densitometry analysis on the levels of p62 (A) and LC3-II (B) from the immunoblots in Figure 7C; N = 3. (C-D) Densitometry analysis on the levels of p62 (C) and LC3-II (D) from the immunoblots in Figure 7E; N = 4. The data is presented as mean ± SEM; ns: not significant, *$p < 0.05$, **$p < 0.01$, ***$p < 0.001$.
